# Glycocalyx regulates the strength and kinetics of cancer cell adhesion revealed by biophysical models based on high resolution label-free optical data

**DOI:** 10.1038/s41598-020-80033-6

**Published:** 2020-12-30

**Authors:** Nicolett Kanyo, Kinga Dora Kovacs, Andras Saftics, Inna Szekacs, Beatrix Peter, Ana R. Santa-Maria, Fruzsina R. Walter, András Dér, Mária A. Deli, Robert Horvath

**Affiliations:** 1 Nanobiosensorics Laboratory, Institute of Technical Physics and Materials Science, Centre for Energy Research, Konkoly-Thege M. út 29-33, 1120 Budapest, Hungary; 2 Institute of Biophysics, Biological Research Centre, Temesvári krt. 62., 6726 Szeged, Hungary; 3 Doctoral School of Biology, University of Szeged, Közép fasor 52., 6726 Szeged, Hungary; 4 Department of Biotechnology, University of Szeged, Közép fasor 52., 6726 Szeged, Hungary

**Keywords:** Motility, Nanoscale biophysics, Applied optics, Optical sensors, Molecular modelling, Glycobiology, Cancer

## Abstract

The glycocalyx is thought to perform a potent, but not yet defined function in cellular adhesion and signaling. Since 95% of cancer cells have altered glycocalyx structure, this role can be especially important in cancer development and metastasis. The glycocalyx layer of cancer cells directly influences cancer progression, involving the complicated kinetic process of cellular adhesion at various levels. In the present work, we investigated the effect of enzymatic digestion of specific glycocalyx components on cancer cell adhesion to RGD (arginine–glycine–aspartic acid) peptide motif displaying surfaces. High resolution kinetic data of cell adhesion was recorded by the surface sensitive label-free resonant waveguide grating (RWG) biosensor, supported by fluorescent staining of the cells and cell surface charge measurements. We found that intense removal of chondroitin sulfate (CS) and dermatan sulfate chains by chondroitinase ABC reduced the speed and decreased the strength of adhesion of HeLa cells. In contrast, mild digestion of glycocalyx resulted in faster and stronger adhesion. Control experiments on a healthy and another cancer cell line were also conducted, and the discrepancies were analysed. We developed a biophysical model which was fitted to the kinetic data of HeLa cells. Our analysis suggests that the rate of integrin receptor transport to the adhesion zone and integrin-RGD binding is strongly influenced by the presence of glycocalyx components, but the integrin-RGD dissociation is not. Moreover, based on the kinetic data we calculated the dependence of the dissociation constant of integrin-RGD binding on the enzyme concentration. We also determined the dissociation constant using a 2D receptor binding model based on saturation level static data recorded at surfaces with tuned RGD densities. We analyzed the discrepancies of the kinetic and static dissociation constants, further illuminating the role of cancer cell glycocalyx during the adhesion process. Altogether, our experimental results and modelling demonstrated that the chondroitin sulfate and dermatan sulfate chains of glycocalyx have an important regulatory function during the cellular adhesion process, mainly controlling the kinetics of integrin transport and integrin assembly into mature adhesion sites. Our results potentially open the way for novel type of cancer treatments affecting these regulatory mechanisms of cellular glycocalyx.

## Introduction

Cell adhesion is a fundamental biological process in which cells can attach to a substrate or to another cell through specific receptor–ligand interactions. Adherent cells rearrange their cytoskeleton to obtain an appropriate contact area with cell type dependent morphology. Cell adhesion receptor molecules play a major role in embryonic development, differentiation, tumor formation, metastasis and cell migration as well^1^. Cell adhesion receptors are categorized according to their protein structure forming the family of integrins, cadherins, immunoglobulins and selectins^2^. Integrins have a prominent role among cell adhesion molecules. Their main function is to establish connections between the extracellular matrix (ECM) and the cytoskeleton. Integrins bind to extracellular ligands and form clusters building up the intracellular focal adhesion complexes^3^. The smallest peptide motif integrins can recognize is the RGD (arginine–glycine–aspartic acid) amino acid sequence. Of the 24 human integrins, eight integrin dimers, i.e., αvβ1, αvβ3, αvβ5, αvβ6, αvβ8, α5β1, α8β1, and αIIbβ3, recognize the tripeptide RGD motif in ECM proteins. These members form the most important integrin receptor subfamily that is important for cancer cells and their metastasis^4^. Integrins specifically interact with RGD motifs initiating the cell adhesion process. This mechanism is highly dependent on the number, position and distance of the RGD motifs on the interface^5–7^. Adhesion receptor expression, the receptor conformational changes, the receptor trafficking in the plasma membrane^8^, integrin clustering, receptor–cytoskeleton interactions—are the major mechanisms that regulates receptor-depended cell adhesion^9^. By employing peptides and peptidomimetics containing the RGD motif, one can create effective integrin ligand displaying particles that inhibit the adhesion of adherent cells to ECM proteins and thereby regulate integrin-mediated tumor biological functions^10^. Importantly, cell adhesion motifs displaying engineered surfaces with tunable biological properties have opened the way to control cell adhesion in both basic and application oriented researches^5,11–15^. Especially, in surface sensitive biosensors these synthetic coatings were found to be useful for controlling cell adhesion in a straightforward manner and to monitor cellular changes due to external stimuli^5,11,16–20^. Cells encounter surrounding surfaces first through their glycocalyx and therefore it may substantially contribute to crucial physiological and pathophysiological processes. The glycocalyx is a carbohydrate-enriched sugar coating that covers the surface of many cells, including cancer cells, presumably greatly influencing cellular interactions with their environment at the molecular scale. Its components are glycolipids, glycoproteins and glycosaminoglycans (GAGs). Glycocalyx contains large amounts of chondroitin sulfate (CS), dermatan sulfate, heparan sulfate, sialic acid, and hyaluronic acid, all negatively charged at neutral pH^21^. Yet it is unclear how the cells reorganize their glycocalyx during adhesion, and how the glycocalyx environment—where adhesion receptors reside and interact—contributes to receptor function.

Enzymatic removal of the glycocalyx components of cancer cells can affect membrane shape^22^ or influence receptor organization and activity^23^. Glycocalyx components chondroitin-4-sulfate, chondroitin-6-sulfate and dermatan-sulfate can be cleaved by enzymes, such as chondroitinase ABC (ChrABC)^24^.

Glycocalyx has an important role in the integrin function and signaling, which is yet unclear in full detail. This relationship can be extremely important in cancer diseases, as the composition or structure of the glycocalyx in approximately 95% of most cancer cells is modified, and because of integrin clustering is functionally associated with loss of tissue homeostasis and the development of a malignant phenotype^25^. Thickness of the glycocalyx is in the range of tens to hundreds of nanometers, depending on cell type and cell states, and it exceeds the length of integrin receptors^26,27^. Moreover, the amount of glycocalyx is significantly higher in cancer cells than in healthy cells^28,29^. Importantly, there is no consensus in the literature on the exact role of cell surface polysaccharides and their derivatives in cellular adhesion. Namely, some works report an increase^30^, while others a decrease in adhesion strength^24,31–33^ when the chondroitinase-digested glycocalyx components are removed or reduced.

Cancer cells are typically adherent cells and thus, adhesion is essential for their survival and metastatic invasion. If we prevent this adhesion, cancer cells die^34^. Consequently, the fine details of cell adhesion regulation are essential for cancer research, but only a small number of methods are suitable for real-time and quantitative monitoring of these processes, especially with high data quality and without introducing any labeling molecules^5^. Recently, it was highlighted by Paszek et al. that novel physical methods, especially in combination with model calculations, will be highly relevant in revealing the exact roles of cancer glycocalyx in the complicated and dynamic process of receptor mediated cellular interactions, including adhesion^23,25,35^.

Cell adhesion can be well characterized by the force received by separating the cell from the substrate or from another cell^16,36^. However, force measuring technologies are typically low throughput methods^16,36,37^. Geometrically, cell adhesion on a flat surface is the transformation of a cell from a spherical object to a flattened, adherent form by maintaining the volume. The progress of adhesion can be also well characterized by the change in cell-substrate contact area^17^. Surface sensitive label-free optical biosensors can be effectively used to study the real-time kinetics of cell adhesion with extremely high resolution^38^. Especially, the evanescent field-based devices are relevant, since they only monitor the cell-substratum contact zone, a 100–200 nm thick layer at the adhering surface of the cells^5,11,39,40^. The measured signal is an integrated response of contact area and molecular density inside the contact zone, an excellent measure of adhesion progression, and was demonstrated to correlate with the adhesion force^11,17,40^. Optical waveguide lightmode spectroscopy (OWLS)^41^ as well as surface plasmon resonance (SPR)^42^ based biosensors are such techniques, however, they have low throughput. This is a disadvantage if simultaneous screening of a large number of samples is needed, and excellent data quality is a must.

Contrarily, the resonance waveguide grating (RWG) technology^5,18,39,43–46^ is available as a high-throughput device with outstanding surface sensitivity and time resolution. Importantly, this technology can measure even single cells and its signal was recently calibrated to adhesion force using fluidic force microscopy^16^. Membrane movements and cell mass redistributions at the nanometer scale are perfectly detectable by the RWG technology^5,44^. Of note, the RWG biosensor is also suitable to study cellular signalization processes in real-time, even in non-adherent cell types^44^. Therefore, the high resolution real-time kinetic data recorded by RWG has every potential to illuminate yet hidden details of cellular adhesion processes at the molecular scale, and supply high quality data for modelling^47–49^.

In the present work the RWG technology is employed to record the adhesion kinetics of HeLa cancer cells while components of their glycocalyx is enzymatically digested by ChrABC. To support the biosensor data, cancer cells were stained with fluorescently labeled lectin or immunolabeled for CS recognizing glycocalyx, and cell surface charge measurements were also employed to measure the time dependent structural changes in HeLa glycocalyx. Our experiments revealed an enzyme concentration dependent regulatory mechanism of cancer cell glycocalyx during the adhesion process. We developed kinetic and static biophysical models and fitted them to the experimental data, illuminating this way the role of glycocalyx in the molecular scale processes controlling adhesion.

## Materials and methods

### Cell cultures and cell adhesion assay buffer

HeLa cells were cultured in tissue culture polystyrene Petri dishes (Sarstedt, Germany) in a humidified incubator (37 °C, 5% CO_2_) in Dulbecco’s modified Eagle’s medium (DMEM), supplemented with 10% fetal bovine serum (Biowest SAS, France), 4 mM l-glutamine (Merck, Germany), 100 U/ml penicillin and 100 μg/ml streptomycin mixture solution (Merck, Germany). Cells were passaged by using 0.05% (w/v) trypsin and 0.02% (w/v) EDTA solution (Merck, Germany). (The protocols for the employed control cell lines can be found in the Supplementary Information (SI)).

Cell adhesion assay buffer was prepared by adding 20 mM 4-(2-hydroxyethyl)-1-piperazineethanesulfonic acid (HEPES) to Hank’s balanced salt solution (HBSS). Cells were brought into suspension by using pre-warmed trypsin–EDTA solution. Trypsin digestion was terminated by medium addition. Harvested cells were washed two times: centrifuged at 200×*g* for 5 min to remove the complete culture medium and cell pellet was re-suspended in 20 mM HEPES HBSS buffer. Cells were then counted in a hemocytometer and diluted to a final cell density of 8000 cells in 25 µl of HEPES HBSS solution.

### Preparation of enzyme solutions

Chondroitinase ABC (ChrABC) enzyme from *Proteus vulgaris* (C2905, Merck, Germany) was used for the digestion of glycocalyx of HeLa cells. Stock solution of the enzyme (4 and 2.5 U/ml) was prepared in 20 mM HEPES HBSS buffer and stored at − 20 °C until use.

### Zeta potential measurements on living cells

To characterize the surface charge density of cells, the zeta potential of HeLa cells was measured before and after ChrABC treatment by the laser-Doppler velocimetry method using the Zetasizer Nano ZS instrument (Malvern, UK). The electrophoretic mobility (μ) of the cells was detected by measuring the Doppler-shift of the backscattered laser light from the cells migrating in a 10 V/cm electric field, and the zeta-potential (${\upzeta }$) was calculated from the Smoluchowski equation (below), where ε is the dielectric constant and η is the viscosity of the suspension: ${\upzeta } = \frac{{4{{\uppi \upmu \upeta }}}}{{\upvarepsilon }}$.

**Figure 1:**
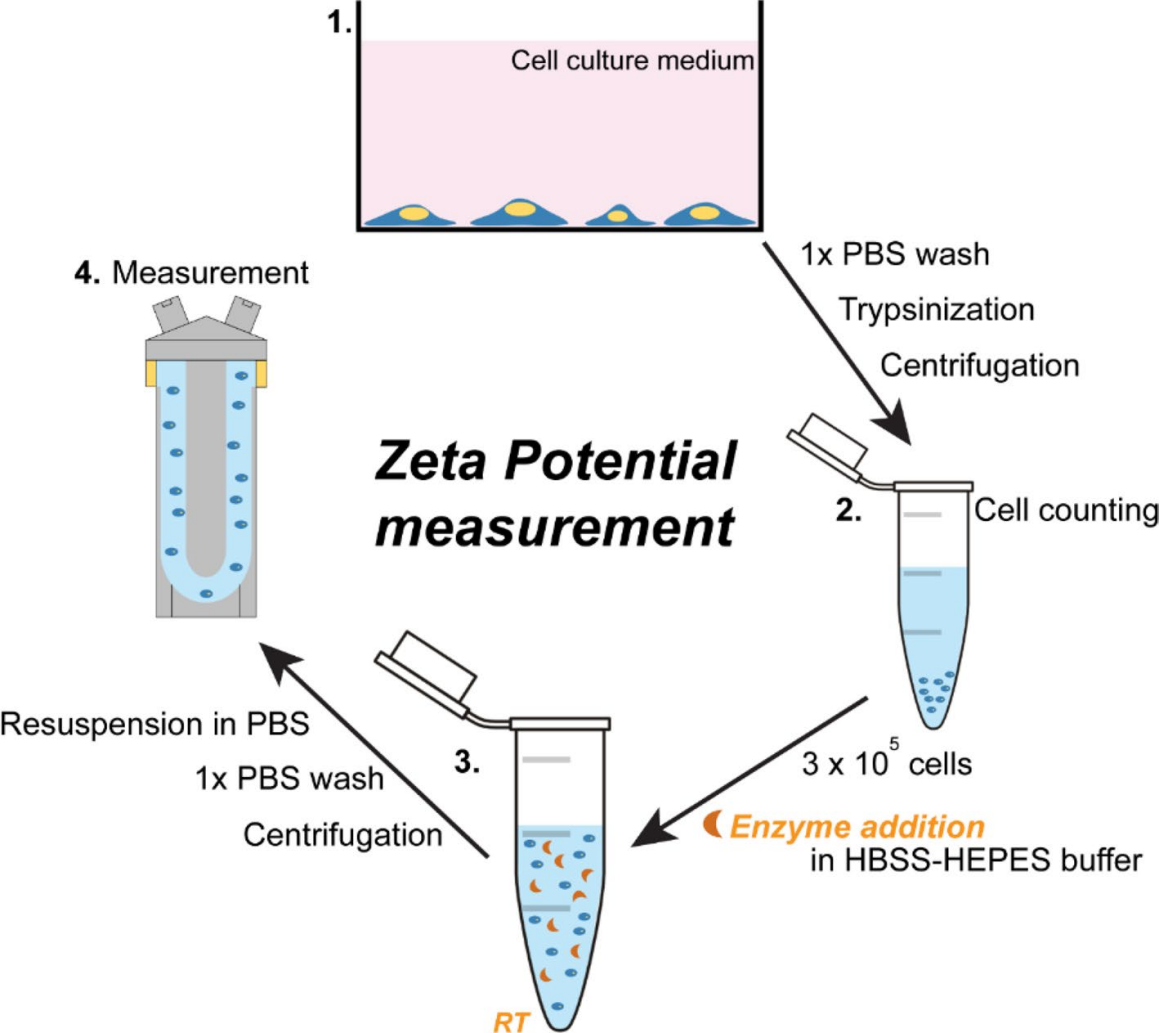
Zeta potential measurement on cells. Cartoon explaining point-by-point the steps of the zeta potential measurement performed on cell suspensions. Adherent cells were collected with trypsin, counted, and treated with ChrABC enzyme in HEPES HBSS buffer. At the end of the incubation time cells were centrifuged, the cell pellet washed with PBS and zeta potential of cells was measured using a Malvern Zetasizer Nano instrument.

A minimum of 6 measurements per sample were made with a maximum of 100 runs each using a disposable zeta potential cuvette with platinum electrodes coated with gold (DTS1070, Malvern, UK)^50,51^. To activate the zeta potential cuvette, it was rinsed once with 100% ethanol and washed twice with distilled water, as described in the manufacturer´s protocol. After electrode activation, the quality of each zeta cuvette was verified using the zeta standard solution (Malvern, UK). Steps of the measurement are visualized in Fig. 1.

First, cells were briefly trypsinized until they rounded up, then trypsin was removed, and cells were collected in phosphate buffered saline (PBS). Subsequently, they were counted, aliquoted to microtubes (3 × 10^5^ cells/vial) and centrifuged, then they were re-suspended in 300 µl 20mM HEPES HBSS buffer containing 1.25 U/ml ChrABC, incubated at room temperature for 30, 60 and 120 min. After each incubation time the treatment was stopped, cells were pelleted by centrifugation, and were re-suspended in 300 µl of PBS with Ca^2+^ and Mg^2+^. For the zeta potential measurement, 10^5^ cells were added to 900 µl of PBS with Ca^2+^ and Mg^2+^. Control cell groups did not receive enzymatic treatment. Control 0 min group was measured directly after cell counting. Control 120 min group was kept in plain HEPES HBSS buffer for 120 min at room temperature similarly to the respective treatment group.

### Chondroitin sulfate immunostaining and confocal microscopy

**Figure 2:**
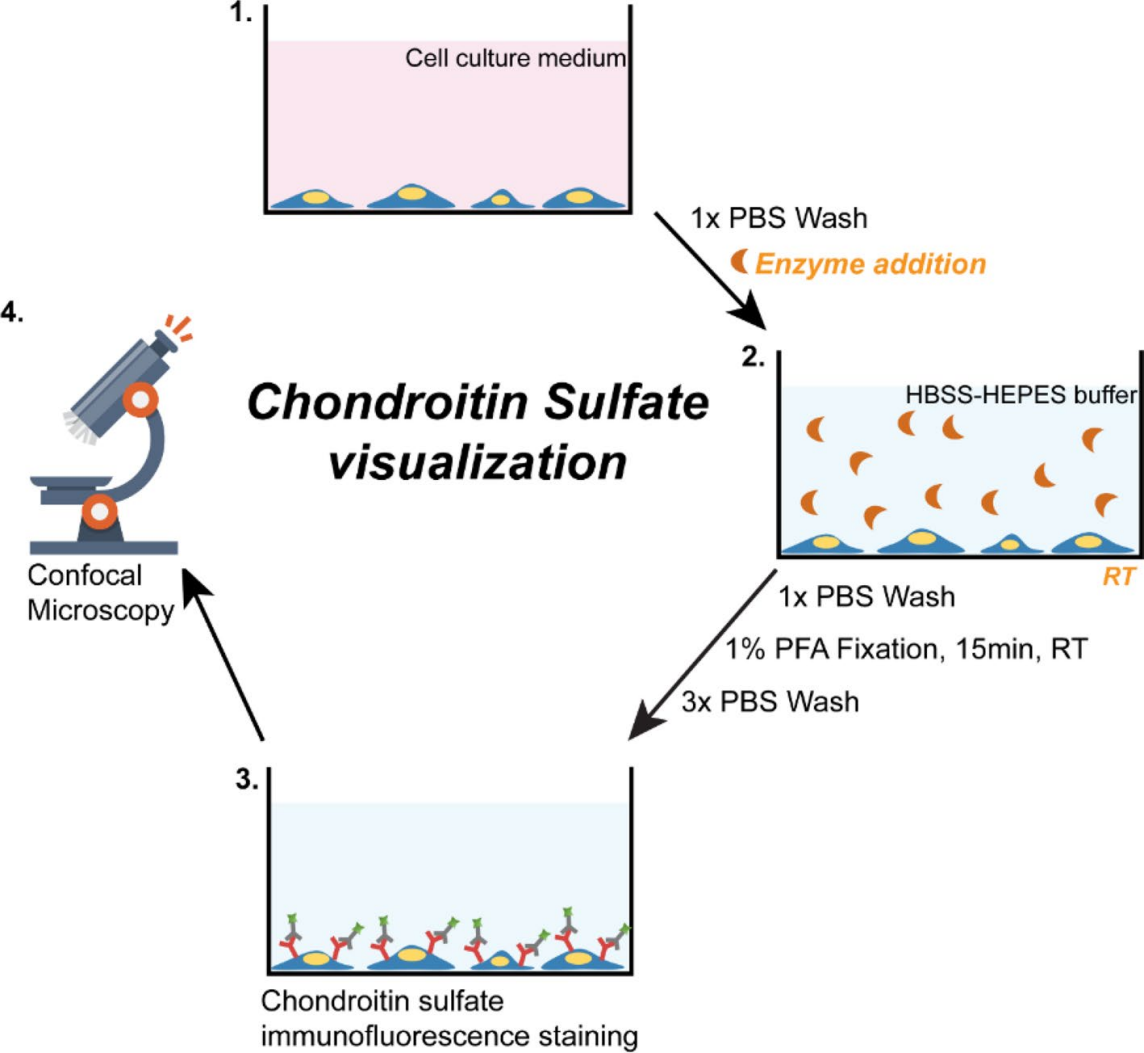
Chondroitin sulfate immunostaining. Cartoon explaining point-by-point the steps of the culture and staining procedure leading to the visualization of surface chondroitin sulfate. After 2–3 days of culture cells were washed with PBS and treated with ChrABC enzyme. After the end of the incubation time cells were washed with PBS, fixed with paraformaldehyde (PFA)-PBS and immunohistochemistry was performed and visualized by confocal microscopy.

HeLa cells were cultured on poly-L-lysine coated glass cover slips for 2–3 days until they covered the surface and treated with ChrABC in HEPES HBSS buffer at room temperature with concentrations between 1.00 × 10^–5^ and 1.25 U/ml for 0 and 60 min (Fig. 2).

After treatment cells were fixed with 1% paraformaldehyde (PFA)-PBS for 15 min at room temperature. Cells were not permeabilized to ensure only surface labeling. Then non-specific binding sites on cells were blocked with 3% bovine serum albumin-PBS for 30 min at room temperature. Chondroitin sulfate was labeled with an anti-chondroitin sulfate mouse monoclonal primary antibody (Merck, Germany; 1:100; AB_476879) overnight in blocking buffer at 4 °C. The next day cells were washed in PBS and incubated with an anti-mouse-Alexa Fluor 488 secondary antibody (Life Technologies, USA, 1:400) for 1 h at room temperature. Cover slips were then washed with PBS and mounted with Fluoromount G mounting medium (Southern Biotech, USA). Pictures were taken with an Olympus FV1000 confocal microscope at random positions, at least 5 images/cover slip. Treatments were performed in triplicates (15 images/group). Fluorescent images were analyzed for staining intensity using the FIJI (ImageJ) software.

### Label-free resonant waveguide grating (RWG) imager biosensor

The Epic BT system (Corning Incorporated, Corning, NY, USA) used is a next generation resonant waveguide grating imager biosensor allowing the high-throughput and label-free detection of living cells. The RWG imager accepts 96- or 384-well *Society for Biomolecular Screening* (SBS) standard format biosensor microplates. In this study 384-well plates (#5040, Corning Incorporated, Corning, NY, USA) were used. The bottom of the Epic microplates consists of a high refractive index optical waveguide layer on top of a glass substrate. The waveguide layer is made of biocompatible material niobium pentoxide. In the center of each well, an optical grating is embedded in the waveguide layer, which functions as tiny sensors. During detection, the gratings are illuminated by a tunable light beam at 825–840 nm. When hitting the so-called resonant wavelength, the grating couples the light into the waveguide layer generating an exponentially decaying electromagnetic field with a penetration depth of 150 nm into the aqueous solution covering the sensors. Any refractive index change inside the evanescent field detunes the resonance and shifts the resonant wavelength. The image of the resonant wavelength is captured by a charge-coupled device (CCD) camera, and the resonant wavelength change in all wells can be monitored in real-time simultaneously with a time resolution of 3 s. The measured final signal is a wavelength shift (Δλ) relative to the given resonant wavelength measured at a reference time (baseline). The wavelength shift is displayed in picometers (pm). Living cells adhering on the biosensors affect the refractive index inside the evanescent field, and consequently shift the resonant wavelength^40,43,48^.

### Polymer solutions for coating the biosensor surfaces

The synthetic copolymers, poly(L-lysine)-*graft*-poly(ethylene glycol) (PLL-*g*-PEG, [PLL(20)-g(3.5)-PEG(2)]) (hereafter PP) and its RGD-functionalized counterpart, PLL-*g*-PEG/PEGGGGGYGRGDSP (PLL-*g*-PEG-RGD [PLL(20)-g(3.5)-PEG(2)/PEG(3.4)-RGD]) (hereafter PPR) and PLL-*g*-PEG-(DBCO-Mal)-CKK-(Acp)-(Acp)-(Acp)-GRGDS (PLL(20)-g(3.5)-PEG(2)/PEG(3.5)-RGD) (hereafter PP-DBCO-R) were obtained as powders from SuSoS AG, Dübendorf, Switzerland.

The materials were stored at − 20 °C until use. Each powder was then dissolved in 10 mM HEPES at pH 7.4 to make stock solutions with a concentration of 1.0 mg/ml and sterile filtered. Coating solution with different concentration of RGD-motifs and PLL-*g*-PEG were prepared by mixing the two 1 mg/ml stock solutions (hereafter PP:PPR).

### Coating protocols to form the RGD density tuned biosensor surfaces

During surface preparation, some wells of the 384-well microplate (#5040, Corning Incorporated, Corning, NY, USA) were hydrated with 30 μl of 10 mM HEPES buffer, pH 7.4 for 20 min and then the buffer was removed. After that, 30 μl of various proportions of PP and PPR were pipetted into the wells. Bubbles were formed during pipetting, so the plate was centrifuged in the Allegra X-30R centrifuge (Beckman Coulter), at 800×*g* for 10 s to eliminate the bubbles, which may greatly affect the measurement. Finally, the plate with desired coating solutions was incubated for 30 min while gently shaking at room temperature.

### Cell adhesion on the biosensor surfaces and subsequent microscopy measurements

The coated wells were washed three times with 50 µl of 20 mM HEPES HBSS. Following the washing steps, 20 µl of ChrABC at different concentrations or 20 mM HEPES HBSS buffer (negative control) was added to the wells with a digital 16-channel Finnpipette Novus (Thermo Fisher Scientific, Waltham, MA, USA) pipette set to stepping mode. After, the plate was inserted into the Epic BT device and a baseline without the cells was recorded. After recording a stable baseline, 20 µl of cell suspension containing 8000 cells in HEPES HBSS buffer was added to the wells using the 16-channel pipette, and cell adhesion kinetics were monitored until reaching saturation. Wells containing buffer for control measurements were also coated with the corresponding copolymers and treated in the same way as the sample wells throughout the experiments, except that they received assay buffer instead of cell suspension. All experiments were done in triplicates in real-time. At the end of the measurement, the microplate was placed under an Axio Observer Z1 inverted light microscope to visualize the cells by using a 20× objective.

### Statistics, data processing and further analysis

Zeta potential measurement data are calculated as means ± standard deviation (SD). Statistical significance between treatment groups was determined using one-way ANOVA with Bonferroni multiple comparison post-tests (GraphPad Prism 5.0; GraphPad Software, USA). The number of parallel samples was minimum three. Changes were considered to be statistically significant at p < 0.05.

The shifts in the resonant wavelength, as the main signals detected in the Epic BT assay, were determined in at least three parallel treatments in each experiment, and all values are presented as mean ± SD. Obtained data were analyzed by using Origin 8.5 (OriginLab Corp., Northampton, MA, USA).

PLL-*g*-PEG surface (100%) was used as a control in all biosensor experiments, owing to its protein-resistant and cell-repellent properties on metal oxide surfaces^11,37,52^. The wavelength shift values recorded on this surface were always subtracted from the data recorded with the RGD displaying surfaces. The obtained kinetic cell adhesion data was fitted with a sigmoidal curve (four parameter logistic regression equation) in Origin 8.5 and it was also with coupled differential equations using a home developed MatLab code.

## Results

### Label-free measurement of cell adhesion kinetics of ChrABC treated cells

**Figure 3:**
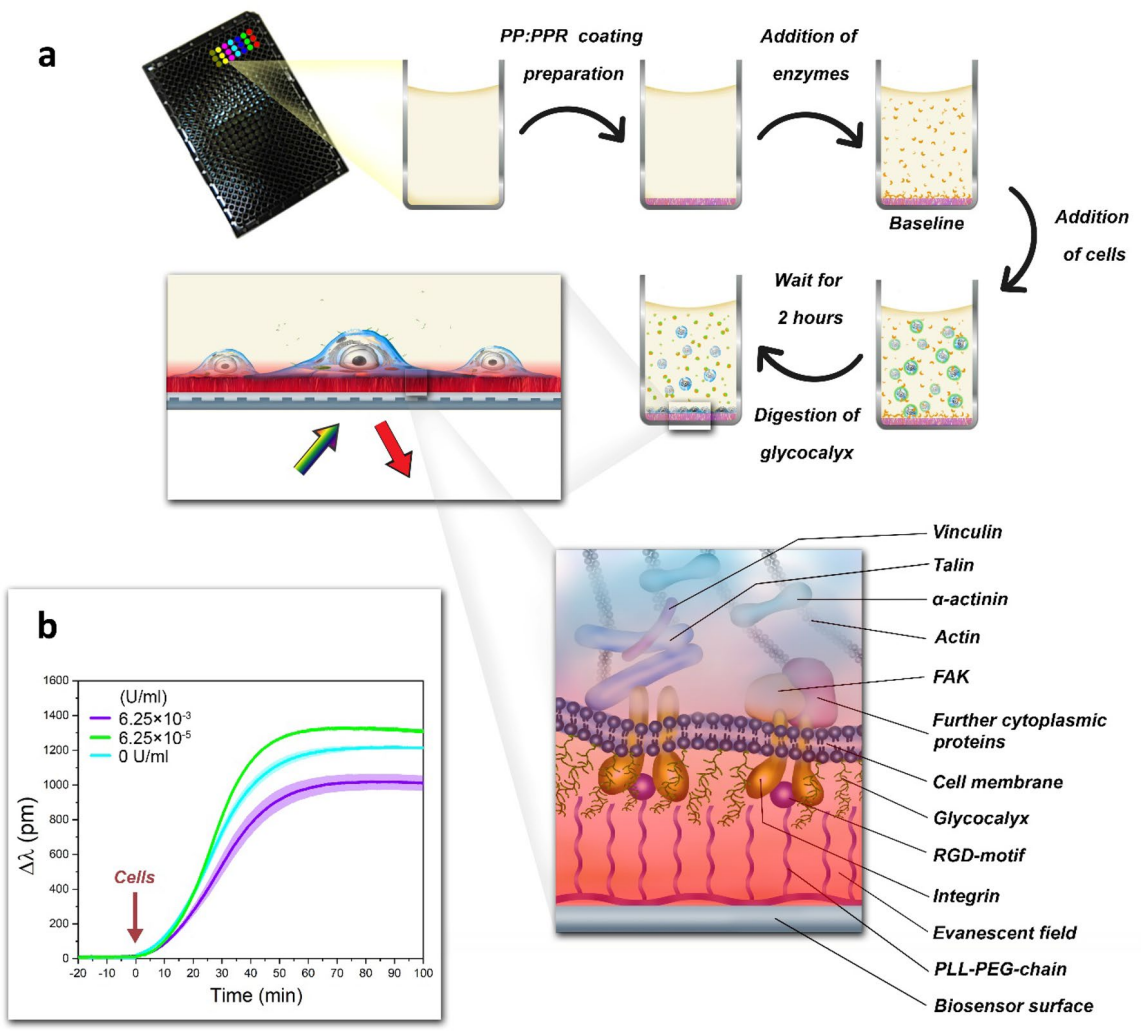
Schematics of RWG measurements of cell adhesion kinetics on the polymer coated biosensor surfaces. (**a**) The adhesion kinetics of cells were real-time monitored using the label-free optical biosensor. First, the PP: PPR copolymer coating was prepared on the sensor surfaces, and the ChrABC enzyme at different concentrations was added to the wells. After recording a baseline HeLa cells were pipetted into the biosensor wells (0 min). The cell adhesion was monitored for 100 min. The schematic illustration of the adhered cells in the biosensor wells and the cellular components are also shown in the magnified parts. The surface localized evanescent optical field is illustrated as red shadow. (**b**) Representative cell adhesion kinetic curves on 50% PP: PPR copolymer surface. Cell adhesion resulted in sigmoidal shaped kinetic curves with varying magnitude, depending on the actual enzyme concentration: 6.15 × 10^−5^ U/ml enzyme increased, while 6.15 × 10^−3^ U/ml decreased the magnitude of the biosensor curves compared to the reference data with 0 U/ml. All the experiments were done at least in triplicates; data are presented as mean ± SD.

The effect of glycocalyx digestion on cell adhesion kinetics was systematically investigated by the RWG sensor in real-time. The steps of the measurements are shown in Fig. 3. First, the wells of the microplate were coated with the polymers and ChrABC were added to the wells. After recording a stable baseline living cell solutions were added to the wells and the kinetics of cell adhesion was monitored. Of note, during the RWG experiments the adhesion of the cells and the digestion of the glycocalyx progressed simultaneously. Cells were always added at 0 min and their adhesion was monitored for 100 min.

Increasing the ChrABC enzyme concentration was found to clearly affect the maximum wavelength shift, that is, the adhesion strength of cells to the surface^16^. Interestingly, the effect was concentration dependent, the enzyme at different concentrations either increased or decreased cell adhesion (Fig. 1b). The high resolution of RWG made possible to detect even tiny differences in the adhesion kinetics, even at exceptionally low enzyme concentration levels.

### Zeta potential and chondroitin sulfate immunostaining of ChrABC treated HeLa cells

**Figure 4:**
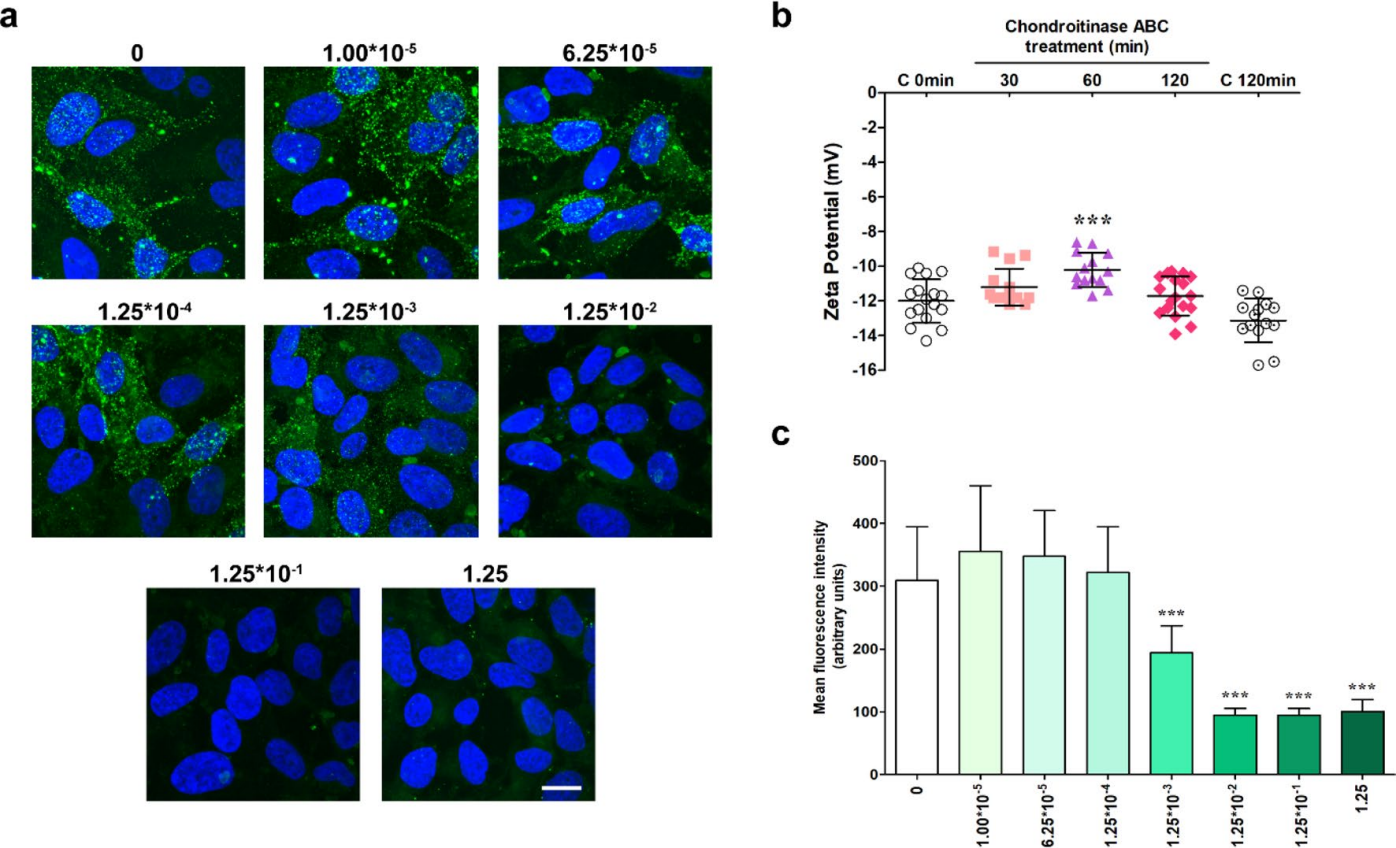
(**a**) Representative pictures of the chondroitin sulfate immunostaining on HeLa cells after 1.00 × 10^–5^–1.25 U/ml ChrABC treatment for 60 min incubation time. Scale bar: 20 µm. (**b**) Zeta potential measurement after 1.25 U/ml ChrABC treatment on HeLa cells. Measurements were performed after 30, 60 and 120 min incubation. Three biological parallels were measured (n = 11–20). ***p < 0.001 compared to the 0 min control. One-way ANOVA with Bonferroni post-test. (**c**) Intensity measurement of the chondroitin sulfate stainings. n = 15 images/treatment groups, ***p < 0.001 compared to the untreated control. One-way ANOVA with Bonferroni post-test.

Initiated by the biosensor data, we measured the consequences of enzymatic digestion on the glycocalyx with two additional techniques at various time points of the digestion process. The effect of ChrABC treatment on the cell surface charge was monitored by the zeta potential of HeLa cells before and after the treatment. Chondroitin sulfate was visualized on HeLa cells by using specific anti-chondroitin sulfate antibody (Fig. 4a)^53^. Chondroitin sulfate staining intensity was quantified by ImageJ (Fig. 4c).

During the zeta potential measurements, cells in suspension were treated with 1.25 U/ml of ChrABC in HEPES HBSS for 30, 60 and 120 min (Fig. 4b). We confirmed that this concentration does not change the viability of Hela cells (see Supplementary Information (SI) Fig. S1). Control (untreated) cells were measured twice: one control group was measured directly after the cell counting at the beginning of the experiment (C, 0 min), while the other control group was incubated in HEPES HBSS for 2 h without any enzymatic treatment (C, 120 min). The basal zeta potential of HeLa cells was − 11.9 mV. ChrABC treatment significantly increased the zeta potential after 60 min treatment to − 10.2 mV, as a clear consequence of the removal of negatively charged glycocalyx component chondroitin sulfate, which is one of the dominant GAG on HeLa cells^54^. At 120 min no difference was seen anymore between treated cells and the 0 min control. Interestingly, the 120 min control group presented a lower zeta potential (− 13.1 mV) than the 0 min control (− 11.9 mV).

We hypothesize that trypsinization results in a partial digestion of the surface glycocalyx by cleaving surface proteins. ChrABC further removes glycocalyx elements, especially chondroitin sulfate and dermatan sulfate containing GAGs. During the 2 h-incubation HeLa cells might produce glycocalyx components by enzymes and regenerate the surface composition, therefore the effect of ChrABC cannot be detected after a certain period of incubation time. This active glycocalyx remodeling may be responsible for the lower zeta potential of the ChrABC-untreated group (Fig. 4b) at the 2 h time point (C, 120 min).

Based on these experiments ChrABC treatment has an optimum for incubation time if the cells are digested in suspension. Importantly, the maximum effect was measured at 60 min, possibly representing a turning point between glycocalyx digestion and remodeling. These results are in concordance with chondroitinase enzyme kinetics data reaching a plateau between 60 and 120 min^55^.

To visualize the action of the ChrABC treatment HeLa cells were treated on cover slips with 1.00 × 10^–5^–1.25 U/ml enzyme for 60 min at room temperature. All cells were successfully stained with the specific chondroitin sulfate antibody (Fig. 4a). We observed a decrease in the intensity at and above the 1.25 × 10^–3^ U/ml concentration indicating the concentration-dependency of the effect (Fig. 4c). We found a time-dependent effect for ChrABC to specifically remove chondroitin sulfate from the surface of HeLa cells: the fluorescent intensity of chondroitin sulfate immunostaining decreased already after 30 min of enzyme treatment, and the effect persisted until the 120 min time point (SI Fig. S2).

To visualize other components of the glycocalyx, HeLa cells were also labeled with wheat germ agglutinin (WGA) lectin (SI Fig. S3) recognizing N-acetylneuraminic (sialic) acid and N-acetyl-d-glucosamine residues of the glycocalyx. We observed that the intensity of the staining did not change at either of the treatment time-points (SI Fig. S3B) indicating that ChrABC treatment reduces the chondroitin sulfate coverage from the cell surface of HeLa cells, but leaves other glycocalyx components less affected. By a 3D analysis of the confocal images we confirmed that the WGA staining can be observed at the cell surface of HeLa cells (SI Fig. S3C).

Additional experiments were performed with another type of cancer cell line, the breast cancer MCF-7, and a non-tumor line, the preosteoblast MC3T3-E1. We observed a decrease in the chondroitin sulfate immunostaining intensity in the MCF-7 cell line after ChrABC digestion, but saw no change in the case of the MC3T3-E1 cells (SI Fig. S4A,B). The cell surface glycocalyx was labeled with WGA staining for both cell types (SI Fig. S4C,D). As in the case of HeLa cells, we did not see any change in the staining intensity after the ChrABC treatment. In addition to staining glycocalyx, zeta potential was also measured in these cell lines. The basal zeta potential of the MCF-7 cells was − 8.6 mV (SI Fig. S5). ChrABC treatment significantly decreased the zeta potential of MCF-7 cells after 60 min treatment to − 9.7 mV suggesting that other glycocalyx components might contribute to a greater degree to the negative surface charge than chondroitin sulfate. The MC3T3-E1 cells showed a negative baseline zeta potential, − 11.7 mV, similar to that of HeLa cells (− 11.9 mV). The ChrABC treatment decreased the absolute value of the zeta potential to − 10.4 mV, which effect was less strong, but close to the reaction of the HeLa cells to the enzyme digestion. This experiment demonstrates that there are big differences in baseline surface charge values between different cell lines depending on their function, origin and most probably glycocalyx composition.

### The magnitude and speed of HeLa cell adhesion are detuned by ChrABC in a concentration dependent manner

**Figure 5:**
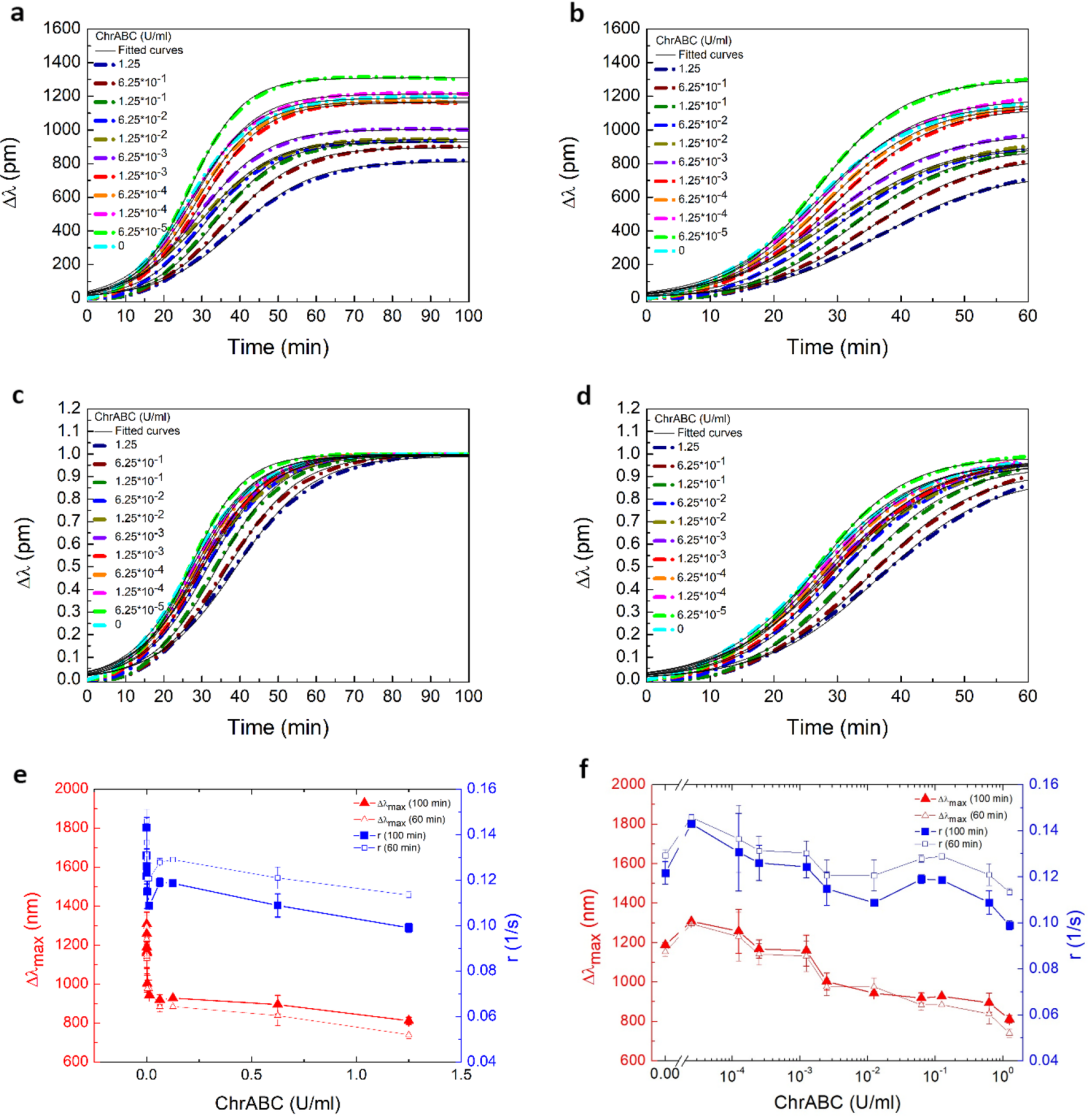
(**a**,**b**) Measured cell adhesion kinetic curves in the presence of ChrABC with 11 different concentrations. The curves were fitted with the logistic equation at the 0–100 min (**a**) and 0–60 min (**b**) time intervals. The fitted black lines are perfectly follow the experimental data. (**c**,**d**) Measured cell adhesion kinetic curves for ChrABC enzyme treatment at various enzyme concentrations normalized to the maximum adhesion signal. The curves were fitted with logistic equation, and their slopes were compared at 0–60 min (**c**) and 0–100 min (**d**) time ranges. (**e**,**f**) Enzyme concentration dependence of ${{\Delta \uplambda }}_{{{\text{max}}}}$ and r parameters obtained from the fits. Both linear (**e**) and logarithmic (**f**) axes are presented. As the fitting was performed both for 0–60 min and 0–100 min time intervals, two data sets are shown for each parameter.

In this section, the real-time adhesion kinetics and its dependence on enzyme concentration are analyzed using RWG data recorded at 11 different ChrABC concentrations. In all experiments cells were adhering on a 50% PP: PPR surface. To better visualize the results, the obtained sigmoidal cell adhesion curves were also normalized to their saturation signal at 100 min. Since maximum glycocalyx digestion was previously measured at 60 min, the kinetic curves were further analyzed in two sets, considering the 0–60 min and 0–100 min adhesion times. The obtained results are shown in Fig. 5.

It is clearly seen already in Fig. 5a,b that low concentrations of ChrABC, namely the 1.25 × 10^–4^ and 6.25 × 10^–5^ U/ml concentrations, resulted in an increased adhesion signal. In contrast, concentrations above 6.25 × 10^–4^ U/ml decreased the adhesion signal. This is already an important finding, suggesting that the cellular glycocalyx has a much more complicated role than a simple protective coat at the surface of the cell, as originally suggested^56,57^. Importantly, the normalized data shown in Fig. 5c,d revealed that the digestion affected not only the magnitude, but the speed of adhesion, the time needed to reach saturation. The slopes of the adhesion curves are clearly depending on the ChrABC concentration, and these differences are well resolved by the biosensor.

To quantify the adhesion curves, the (1) logistic differential equation was fit to the experimental data, from which the $\Delta \lambda_{{{\text{max}}}}$ and *r* values of the kinetic curves were obtained.

1 $$\frac{{{\text{d}} \Delta \lambda }}{{{\text{dt}}}} = r \times \Delta \lambda \times \left( {1 - \frac{ \Delta \lambda }{{ \Delta \lambda_{{{\text{max}}}} }}} \right)$$

$\Delta {\uplambda }_{{{\text{max}}}}$ is the maximum biosensor signal reached at saturation (in pm) and r is the adhesion rate constant (in 1/s) characterizing the speed of adhesion. Note, the integral form of this equation can be found in ref.^17^.

Even if this differential equation has only two free parameters, it fitted all of the data perfectly. Of note, the observed sigmoidal kinetics in Fig. 5a–d is a typical feature of receptor-mediated cell adhesion, and the shape of these adhesion curves correlate with the viability status of the cells^5,11^.

The concentration dependence of the fitting parameters is shown in Fig. 5e,f. Increasing the enzyme concentration seems to sharply decrease the $\Delta \lambda_{max}$ and *r* values form around a value of 1300 pm and 0.14 1/s to 900 pm and 0.11 1/s, respectively. The length of time period used for fitting (0–60 min and 0–120 min) only slightly affected the results, which we believe is due to the fact that all of the adhesion curves almost reach their saturation values at 60–70 min.

**Figure 6:**
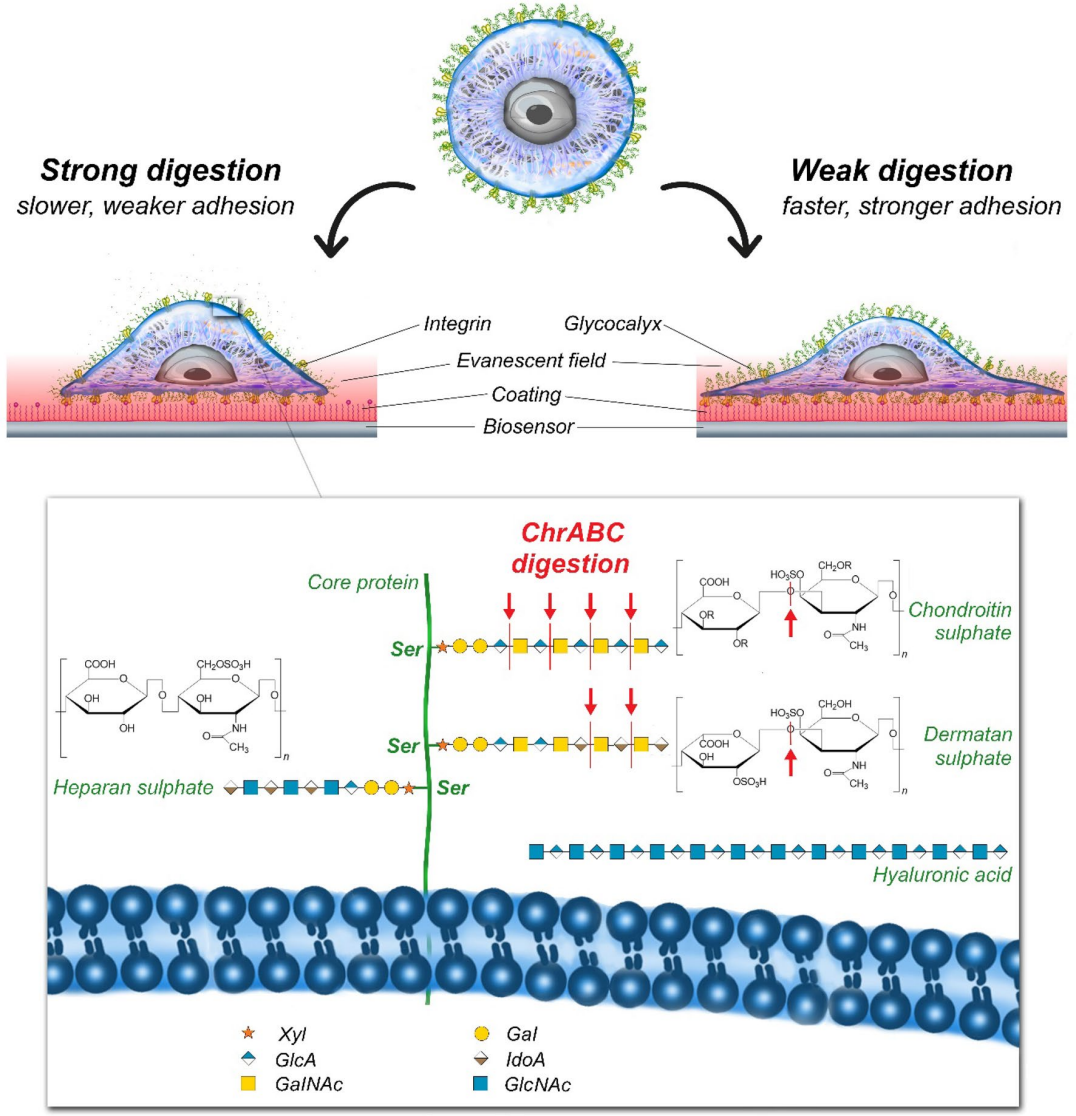
Schematic representation of the effects of cellular glycocalyx digestion by ChrABC on HeLa cell adhesion speed and strength. In general, ChrABC digests O-linked chondroitin sulfate-like glycosaminoglycan^58^. It catalyzes the eliminative degradation of polysaccharides containing (1–4)-β-d-hexosaminyl and (1–3)-β-d-glucuronosyl (or (1–3)-α-l-iduronosyl) linkages to disaccharides containing 4-deoxy-β-d-gluc-4-enuronosyl groups. It cleaves dermatan sulfate, chondroitin 4-sulfate and chondroitin 6-sulfate, but it can act slowly on hyaluronate as well^49,59,60^.

Plotting the obtained results on a logarithmic scale (see Fig. 5f) illuminates that while the overall decreasing tendency in both parameters with increasing enzyme concentration is clear, low concentrations (1.25 × 10^–4^ and 6.25 × 10^–5^ U/ml) increased both the maximum signal and rate of adhesion compared to control. The above results are graphically illustrated in Fig. 6 where the digested glycocalyx components are also shown.

In order to better illuminate the above results, additional experiments were also performed with a breast cancer cell line MCF-7 and the preosteoblast MC3T3-E1 cells. The effect on adhesion was completely missing for the MC3T3-E1 cell line, and was less significant for the MCF-7 cancer cell line (see SI Figs. S6–S8).

At the molecular scale one can assume various scenarios which could explain the above observations. First, (i) the glycocalyx might play a role in the process how the integrins became active, (ii) the glycocalyx components might be necessary to facilitate integrin clustering, and (iii) it cannot be excluded that the glycocalyx plays a role in the transport of integrins into the adhesion zone of the cells. Moreover, (iv) glycocalyx components can be important in the ligand binding process of the integrins and the (v) digestion of glycocalyx can affect the probability that the integrin-ligand complexes fall apart. Furthermore, (vi) the increased adhesion strength and speed with mild digestion of glycocalyx might suggest a simple barrier function which prevents the receptor-ligand binding at certain glycocalyx densities.

### Dynamics of focal adhesion formation: kinetic modeling of cell adhesion data

**Figure 7:**
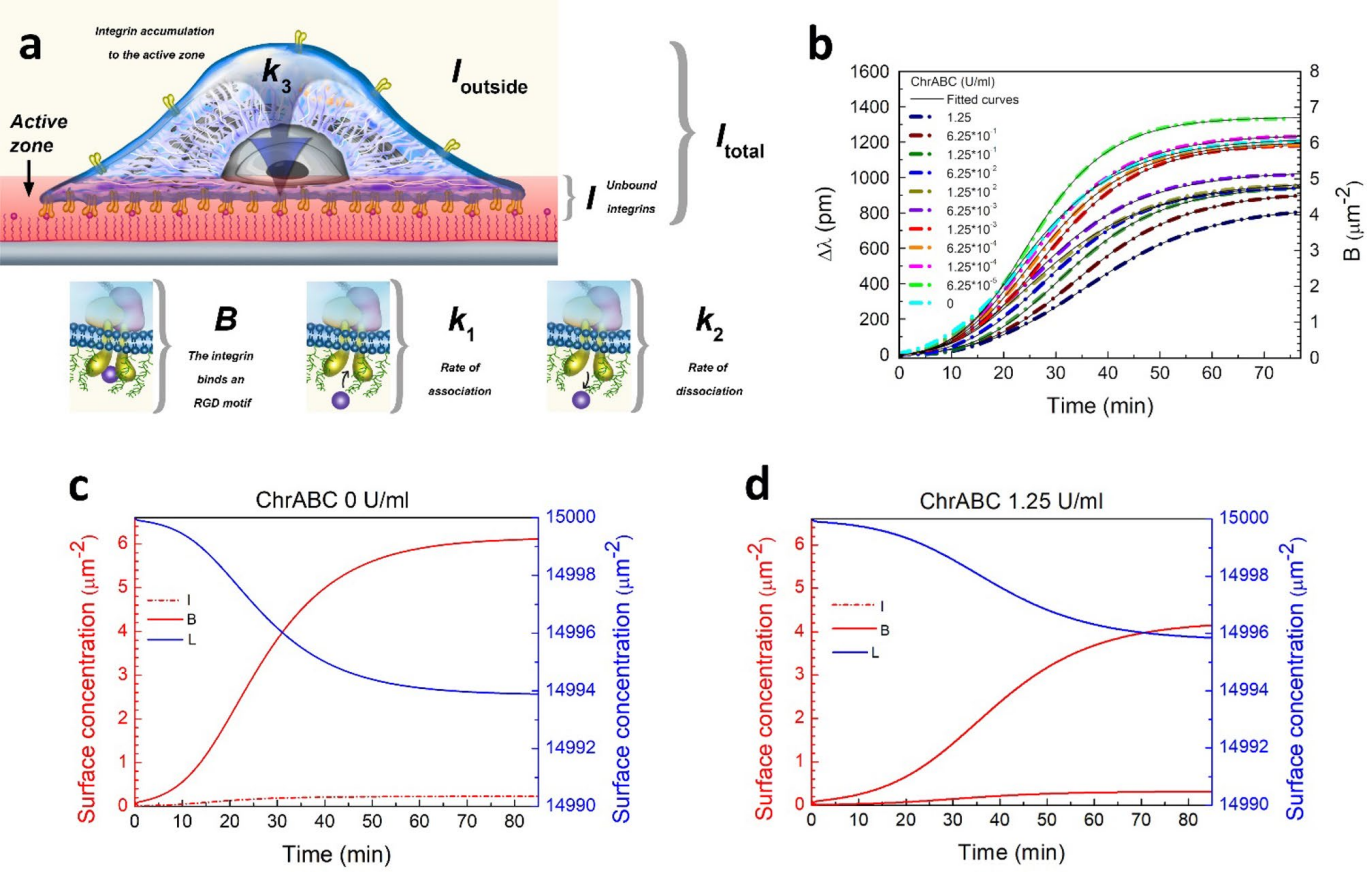
(**a**) Illustration of model parameters determining the adhesion kinetics. It represents the surface concentration of free integrins in the adhesion zone (monitored by the red shadow evanescent field). In the active zone, there are integrins which bind RGD motifs, with surface concentration B. The I and B together provide the surface concentration of all integrins inside the adhesion zone. Outside of the evanescent field (outside of the the adhesion zone), there are also integrins which did not accumulate to the active zone (‘*I*_outside_’). The total number of integrins (‘*I*_total_’) is the sum of the integrins in the active zone (‘*I*’ and ‘*B*’) and the integrins outside the zone (‘*I*_outside_’). *k*_1_ represents the rate of integrin-RGD association, while *k*_2_ the rate of dissociation, and *k*_3_ the rate of the integrin accumulation to the active zone. (**b**) The measured experimental curves at 11 different ChrABC concentrations with the dotted line. The continuous lines represent results of kinetic modelling fitted to the data. (**c**,**d**) The fitted values of integrin (I), RGD (“Ligand”, L) and bound integrin (**b**) surface concentrations in time in case of 0 and 1.25 U/ml enzyme concentrations.

In order to highlight the most important features of the adhesion process at the molecular scale, we have developed a kinetic model taking into account the most relevant biomolecular interactions during cell adhesion. The model considers the ligand binding and dissociation of integrins, and the transfer of integrins into the adhesion zone with simple kinetic rate constants in coupled ordinary differential equations. We constructed equations describing the change in the ligand (*L*), unbound integrin (*I*), and integrin-ligand complex (*B*) surface concentration in the adhesion zone over time:

2 $$\frac{dB}{{dt}} = k_{1} L \times I - k_{2} B,$$

3 $$\frac{dL}{{dt}} = - k_{1} L \times I + k_{2} B,$$

4 $$\frac{dI}{{dt}} = - k_{1} L \times I + k_{2} B + k_{3} B\left( {I_{{{\text{max}}}} - I} \right),$$

where $k_{1} , k_{2}$ are the on–off binding rates of the integrins and their ligands, $k_{3}$ is the recruitment rate of the integrins to the active zone $. I_{{{\text{max}}}}$ is the the maximum possible surface concentration of the integrins in the adhesion zone. Simply, if the integrin concentration reaches $I_{max}$, the recruitment of the integrins stops. Figure 7a summarizes the molecular scale events accounted by our kinetic model.

In order to fit the above kinetic model to the experimental data, first, a calibration between surface concentration (µm^−2^) and wavelength shift signal (pm) must be established. We recorded around 1200 pm wavelength shift at saturation without enzyme treatment. This means that at this wavelength shift value the integrins are at their maximum density at the bottom surface of the cell. From microscopic images we concluded, the averaged HeLa cell at these conditions covers approximately an area of 1000 µm^2^. We estimated 6000 bound integrins on an area of 1000 µm^2^ at saturation^5^ which means that 1200 pm is equivalent to an integrin surface concentration of 7 µm^−2^. Therefore, assuming that the measured wavelength shift is proportional to B^5,19^, the conversion between wavelength shift and surface concentration of bound integrins is $1 {\text{ pm}} \Leftrightarrow \frac{1}{200} {\upmu {\text{m}}}^{ - 2}$.

We developed a MatLab code to fit the experimental data shown in Fig. 7b with the model equations (Eqs. (2)–(4)). We performed the fitting using $k_{1} ,{ }k_{2} ,{ }k_{3}$ and *I*_max_ as fit parameters from the starting point of each sigmoidal curve (t = 0) to its maximum of the three parallel measurements of each ChrABC concentration of the measurement shown in Fig. 7b. The initial surface conditions set were: L(t = 0) =15,000 ${\upmu {\text{m}}}^{ - 2}$ RGD ligands on the surface, B(t = 0) = 0 bound integrin-ligand complex and I(t = 0) = 0.1 ${\upmu {\text{m}}}^{ - 2} { }$ integrins on the bottom of cell when it first gets in contact with the surface. The measured curves and their fitted counterparts are shown in Fig. 7b. It is important to note, our model fits the measured curves with exceptional accuracy (${\text{R}}^{2} > 0.99$).

In Fig. 7c,d not only the surface concentration of the bound integrin-ligand complexes in the adhesion zone are shown, which is proportional to the RWG signal, but also the other two variables of our model: the surface concentrations of the unbound integrins and their free ligands inside the active zone.

**Figure 8:**
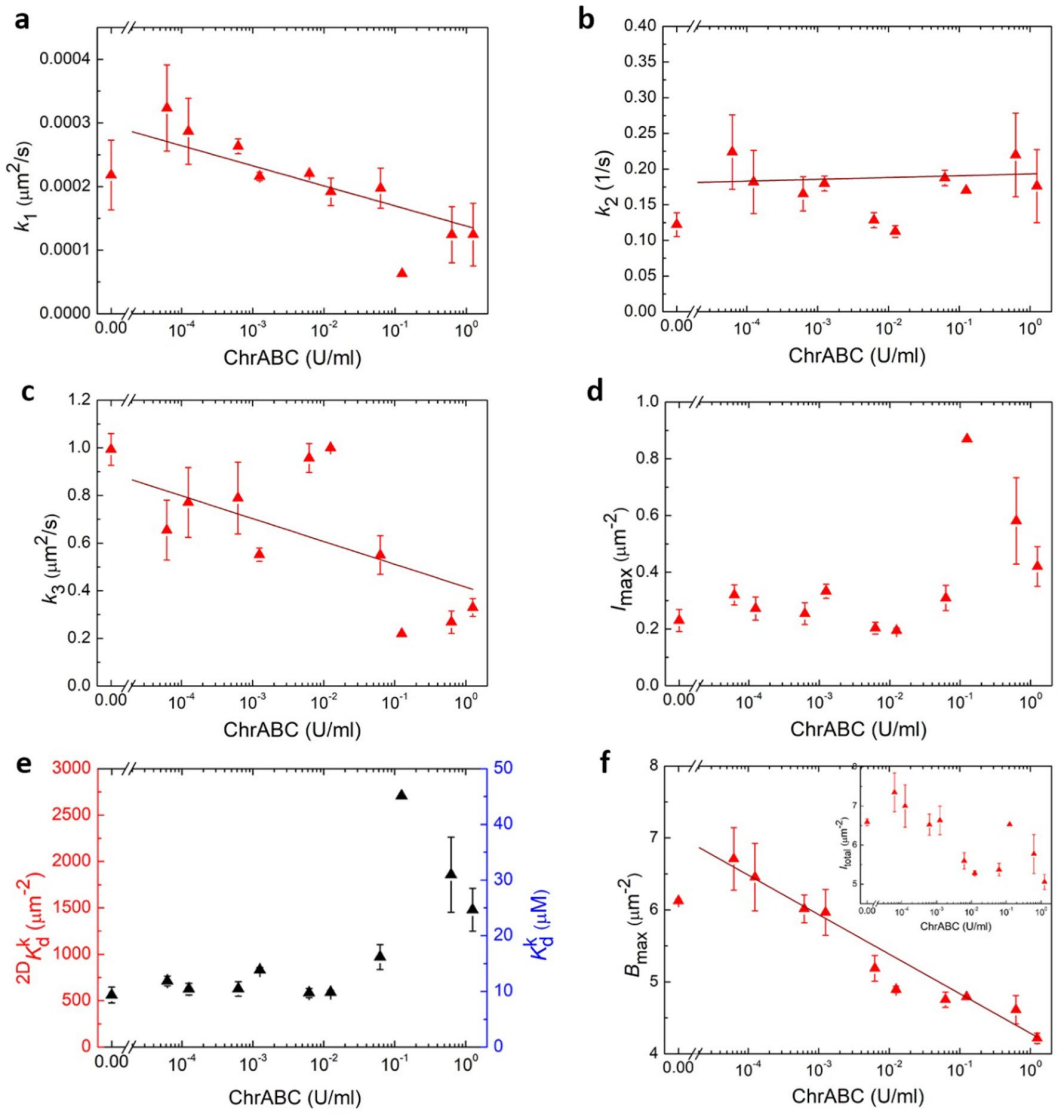
(**a**–**d**) The resulted $k_{1} ,{ }k_{2} ,{ }k_{3}$ and $I_{{{\text{max}}}}$ parameters from the model fitted to the the data at 11 enzyme concentrations. (**e**) The 2D kinetic dissociation constant, ${}_{{}}^{{2{\text{D}}}} K_{{\text{d}}}^{{\text{k}}}$, and the 3D “kinetic” dissociation constant, $K_{{\text{d}}}^{{\text{k}}}$, calculated using the kinetic parameters from the fit. (**f**) Bound integrin-ligand complex surface concentration at saturation, and in Inset: the total number of integrins at saturation, in the function of enzyme concentration.

The enzyme concentration dependence of the fitted kinetic parameters is plotted in Fig. 8. Our analysis shows the ChrABC enzyme treatment decreases integrin-ligand association above 6.25 × 10^–4^ U/ml (decreasing *k*_1_ with increasing ChrABC concentration, see Fig. 8a). However, at the lowest concentrations (6.25 × 10^–5^ and 1.25 × 10^–4^ U/ml) *k*_1_ is increased compared to the control without digestion. Therefore, mild digestion of glycocalyx facilitates integrin-RGD binding. In contrast, the recruitment rate of the integrins, *k*_3,_ decreases with increasing enzyme concentration (Fig. 8c). We found that ChrABC treatment does not affect the dissociation of the integrin-ligand complexes (almost constant *k*_2_, see Fig. 8b). The *I*_max_ parameter is almost constant (0.3 µm^−2^) until 0.01 U/ml concentration, and slightly increases by a factor of two for larger concentrations (see Fig. 8d). This suggests that with intense removal of glycocalyx components the cells tolerate a higher unbound integrin concentration inside the adhesion zone.

From the on–off rates, $k_{1} {\text{and}} k_{2}$, we calculated ${}_{{}}^{2D} {K_{d}^{k}} = \frac{{k_{2} }}{{k_{1} }}$, thereafter the “kinetic” 2D dissociation constant. It is almost constant with increasing enzyme concentration, but starts to increase from around 600 µm^−2^ to values around 1700 µm^−2^, above 0.1 U/ml concentration. (Fig. 8e).

To be able to compare the obtained dissociation constant with other experimentally measured values, the traditional 3D (in solution) dissociation constant should be determined. $K_{d}^{{k}{}}$ is readily obtained by dividing the 2D dissociation constant by the average cell–substrate separation distance (*d*_c_) (the thickness of the confinement zone) according to Eq. (5)^5,61^.

5 $${K_{d}^{s}} = \frac{{ ^{2D} Kd}}{{d_{C} }}$$

Various techniques have been used in the literature to determine (*d*_c_), such as surface plasmon resonance microscopy^62^. The average values obtained are typically in the range of 40–160 nm. Based on these values, a separation distance of 100 nm was employed. The results of calculations are also shown in Fig. 8e. Note, due to the above conversion, the concentration dependence of ${K_{d}^{k}}$ and ${}_{{}}^{2D} {K_{d}^{k}}$ is the same.It is important to note that a larger dissociation constant means weaker binding. Therefore, the remarkably similar trends in ${K_{d}^{k}}$ and *I*_max_ (see Fig. 8d,e) is understandable, presumably the cells try to compensate the weaker binding with a larger amount of free integrins recruited into the adhesion zone.

It is also revealing to investigate the steady state, saturation values of *B*. The concentration dependence of *B*_max_ is shown in Fig. 8f. Increasing ChrABC concentration decreases the saturation level integrin-RGD concentration, the glycocalyx therefore clearly facilitates the formation of integrin-ligand complexes. However, again, at low concentrations the effect is the opposite, *B*_max_ is significantly increases compared to the control. Mild reduction of glycocalyx components induces the formation of more adhesion sites, increasing adhesion strength.

One possible interpretation of the introduced $I_{max}$ parameter is the concentration of maximally available integrins outside of the surface adhesion zone. Therefore, Eq. (4) means the recruitment of the integrins stops when the integrin number in the active zone equals to the number of integrins outside of the surface adhesion zone. (Note, using this interpretation, *I*_outside_ $= I_{{{\text{max}}}} {\text{ in }}$ Fig. 7a). We can then calculate the total integrin surface concentration available, $I_{total} = B + I +$ $I_{max}$, shown at saturation in the inset of Fig. 8f. Based on this interpretation another effect of the enzyme is that it reduces the total number of integrins in the cell potentially available for ligand binding. Again, for low concentrations the effect is opposite, more integrins are available for binding.

It is important to note that while the concentration dependent tendencies of the kinetic parameters are clear, the absolute values are sensitive to the initial parameters employed during the fitting of the model (such as the initial *k*_1_, *k*_2_, *k*_3_ and $I_{outside}$, *k*_1_ being the most critical). We could change the value of the initial *k*_1_ more than two orders of magnitude without significantly increasing the error of the fit. This resulted in a ± 50% deviation in the fitted value of *k*_1._ We could observe only a ± 5–15% deviation in the other three parameters while changing these parameters’ initial values by one order of magnitude. Tuning the initial parameters in a reasonable range, without increasing the error of the fit, the ${K_{d}^{k}}$ value can be shifted by 100%, because it is strongly dependent on the value of *k*_1,_ while, importantly, the relative effect of the enzymatic digestion remained the same.

Based on our findings, we can safely conclude that the glycocalyx has a regulatory role in integrin-ligand binding and also plays a role in the recruitment of the integrins, in their transition to become active. The effect of glycocalyx can be positive or negative, strongly depending on the actual digestion level. Importantly, according to our results, the glycocalyx does not affect the dissociation of the integrin-ligand complexes. This is reasonable, since the glycocalyx components are completely missing or much less dominant building blocks of the mature adhesion sites where the integrins form clusters and are in relatively close contact^23^.

### Static modelling of saturation level adhesion data on RGD tuned surfaces

Our above kinetic modeling of cell adhesion data is a novel way to determine the rate and dissociation constants of integrin-ligand binding, directly in live cells, and without employing any labelling. Note, traditional techniques usually employ isolated integrins to determine the dissociation constant^63^. This is clearly not easily feasible in a complex system, where the effect of other components, such as the building blocks of glycocalyx should be taken into account. Alternatively, the dissociation constant can be obtained from a static measurement using a simple 2D model and saturation level adhesion data recorded on RGD density tuned surfaces^5,11,19^.

In this set of experiments, first, biosensor coatings with various RGD surface densities were fabricated using mixed solutions of PP and PPR. The surface adsorbed polymer mass can be calculated from the raw wavelength shift data based on a calibration equation^40,64^, and the final RGD surface density of the coating is obtained from^5^.

6 $$L_{0} = \frac{\Gamma }{{M_{PPR} }}\frac{Q}{100}\frac{{N_{Lys} }}{g}\frac{P}{100},$$

where Γ = 97 $\frac{{{\text{ng}}}}{{{\text{cm}}^{2} }}$ is the measured density of adsorbed polymer mass, M_PPR =_ 107.76 kDa is the molecular weight of the RGD-functionalized copolymer, Q is the volume ratio of PPR solution in the mixed solution of copolymers (100% means pure PPR solution), N_Lys_ = 136.82 is the average number of lysine monomers in the PLL backbone, g = 3.5 is the grafting ratio (giving the number of Lys units per PEG side chain), P = 14.7% is the fraction of functionalized PEG chains. From *L* the averaged distance between the RGD motifs (d_RGD–RGD_) can be also calculated^19^. Table 1 summarizes the obtained layer parameters for the various mixed solutions used for coating the biosensor wells.

**Table 1:** Parameters of the fabricated RGD displaying surfaces using mixed PP:PPR solutions.

Q (%)	$L_{0} \left( {\frac{{{\text{pmol}}}}{{{\text{cm}}^{2} }}} \right)$	$d_{RGD - RGD}$ (nm)	L_0_ $\left( {\frac{1}{{{\upmu {\text{m}}}^{2} }}} \right)$
0	0.00	∞	0
1	0.05	61.00	306
4	0.21	30.50	1225
10	0.52	19.29	3063
25	1.29	12.20	7657
38	1.97	9.90	11,639
50	2.59	8.63	15,314
58	3.00	8.01	17,765
66	3.41	7.51	20,215
75	3.88	7.04	22,971
80	4.14	6.82	24,503
85	4.40	6.62	26,034
90	4.65	6.43	27,566
95	4.91	6.26	29,097
100	5.17	6.10	30,629

**Figure 9:**
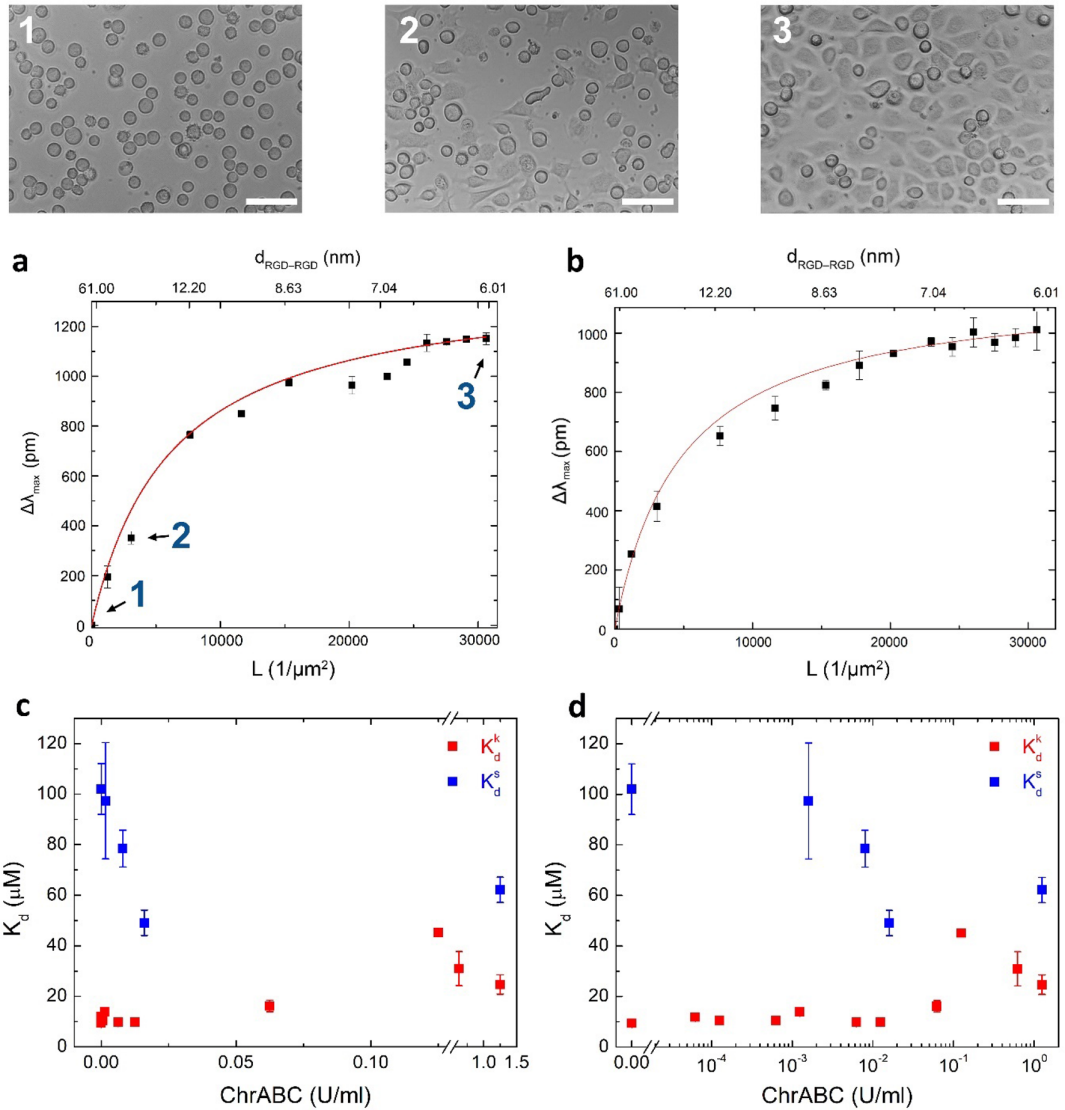
(**a**) Saturation level biosensor data of cell adhesion measured on the RGD-tuned surfaces without enzyme treatment. Microscope images represent adhered cells at three different ligand densities. (Scale bar: 100 µm). The continuous line represents the fit of the employed receptor-ligand binding model (see text for details). (**b**) Maximum wavelength shift as a function of average number of ligands per unit area when HeLa cells were treated with 0.008 U/ml ChrABC and adhered on the surfaces. (The continuous lines represents the fit of the employed receptor-ligand binding model (see text for details). (**c**) Comparison of the obtained “static” and “kinetic” dissociation constants of integrin-RGD binding measured at various enzyme concentrations.

After, the saturation level adhesion signals were recorded on the fabricated RGD tuned surfaces. Increasing the RGD surface density (decreasing the RGD-RGD distance) increases the saturation level adhesion signal (see Fig. 9a,b).

To interpret the saturation data, we assumed that the receptor–ligand interaction could be described as a first-order monovalent binding. The following reaction scheme can be employed:

7 $$L + I \begin{array}{*{20}c} {\mathop \leftrightarrow \limits^{{k_{1} }} } \\ { k_{2} } \\ \end{array} B$$

In steady state, the rate of integrin-RGD association and dissociation are the same. This equilibrium can be described by Eq. (8) if L_0_ >  > B_eq_

8 $$B_{{{\text{eq}}}} = \frac{{L_{0} I_{0} }}{{L_{0} + ^{2D} Kd }},$$

where $L_{0} = L + B$ means the total number of ligands, $I_{0} = I + B$ the total number of integrins, and ${}_{{}}^{2D} {K_{d}^{s}} = \frac{{k_{d} }}{{ k_{a} }}$ is the “static” 2D dissociation constant.

Assuming that $B_{{{\text{eq}} = }} B_{{{\text{max}}}}$ is directly proportional to the optical response at saturation (Δλ_max_)^5,19^, we fitted Eq. (8) to the Δλ_max_—$L_{{}}$ data, obtaining ${}_{{}}^{2D} {K_{d}^{s}}.$ Using then Eq. (5), and the data in Fig. 9a the “static” 3D (the traditional solution) dissociation constant without enzymatic digestion was determined, ${K_{d}^{s}} = 102$ ± 10 ${\upmu {\text{M}}}$.

The above measurements and calculations were also performed on the enzyme treated cells using 10 different ChrABC concentrations. Figure 9b represents the Δλ_max_ − $L_{{0}}$ curve of 0.008 U/ml ChrABC-treated cells. In this case ${}_{{}}^{2D} {K_{d}^{s}} =$ 4708 ± 599 ${\upmu {\text{m}}}^{-2}$ was obtained, which resulted in ${K_{d}^{s}}$ = 78.47 ± 10 ${\upmu {\text{M}}}$.

The measured “kinetic” and “static” dissociation constants measured at various ChrABC enzyme concentrations is compared in Fig. 9c,d we obtained that the two types of dissociation constants are differ by a factor of 10 at low concentrations, but take up similar values at higher ChrABC concentrations.

## Discussion

Integrins are transmembrane proteins, they bind extracellular ligands and initiate the adhesion and migration of cancer cells. A possible regulator of tumor–extracellular matrix interactions is the glycocalyx, a thick coat of sugar-decorated proteins and lipids on the outer surface of all eukaryotic cells. The structure of glycocalyx is heavily altered in cancer cells, but its exact role is not yet understood. This is possibly due to the difficulty of investigating such complicated and dynamically changing biomolecular assemblies at the nanometer scale in real-time, directly inside the living cells.

In our experiments, a highly sensitive, label-free RWG biosensor was applied to monitor the real-time kinetics of HeLa cell adhesion with excellent time resolution and unprecedented data quality. The RWG technique monitors structural changes in the 150 nm thick vicinity of the surface, illuminating this way the time dependent changes in the adhesion zone. The sensor surfaces were coated with an RGD-motif-displaying PP: PPR film with controlled RGD surface densities, meaning different RGD-RGD average distances.

We employed the ChrABC enzyme to digest specific glycocalyx components during the adhesion event. ChrABC mainly digests the chondroitin sulfate components of the glycocalyx^21,54,65^. Lee et al. digested a TE-1 cell line with 1 U/ml ChrABC. They concluded that although it is possible that such an enzymatic treatment removes antiadhesive and adhesive molecules, their data suggest that the net effect of chondroitinase ABC treatment is to increase cell adhesion and increase adhesion strength^30^. In contrast, Moyano digested Human T cells with 1 U/ml ChrABC, which found that adhesion was reduced at similar enzyme concentrations because integrins could not bind to the fibronectin ECM^31^. Chondroitinase ABC had a minor effect at 0.3 U/ml (10% inhibition) and produced 60% inhibition at 1 U/ml^65,66^. Iida et al. also found decreased cell adhesion of ChrABC. 0.1 and 0.01 U/ml enzymes were used on breast cancer cells. It has been suggested that CS components affect cell stability, thereby affecting ligand binding and/or clustering. In their paper, they report that integrins bind directly to CS and that the integrin subunit contains at least one CS binding site. Thus, ChrABC may regulate cell adhesion by integrins^32,33^. Future studies may successfully explore this issue using integrin antibodies.

Note, ChrABC can also digest at high concentrations the hyaluronic acid chains^67–71^. We think that the main point in our work is the cleavage of the glycocalyx elements and how this affects cancer cell attachment and the validity of our observation is not less even if a decrease in HA also happens in addition to CS removal. Importantly, HeLa cell contains the polyanions heparan sulfate and CS^54^, but hyaluronic acid is not present on its surface^72,73^. Nonetheless, in future work, investigating hyaluronic acid of HeLa cells might prove important.

The efficiency of digestion was proven by zeta-potential measurements, reporting on significant surface charge density changes of the enzyme-treated cells. We found that enzymatic treatment of cell suspensions and adherent HeLa cells for different incubation times results in a temporary decrease of the surface zeta potential. To maximize the effect, the optimal incubation parameters were determined, and adapted in the subsequent adhesion experiments. To reveal dynamic changes in glycocalyx during the enzymatic treatment, we also used chondroitin sulfate immunostaining to detect the specificity and efficacy of ChrABC treatment. We noticed a concentration and time dependent reduction in the staining intensity. At the highest treatment concentration (1.25 U/ml) already 30 min treatment resulted in the removal of cell surface chondroitine sulfate, which persisted till the 120 min treatment time. Meanwhile we did not observe any change for WGA staining, which visualizes sialic acid residues on other components of the glycocalyx, like glycoproteins. Overall, effective digestion levels were reached after 30 min of incubation and persisted up to 60 and 120 min, which fall into the time span of the RWG experiments (0–100 min). It should be also noted that plasma proteins protect the endothelial glycocalyx and their depletion may lead to loss of glycocalyx coverage^74,75^. In the work of Zeng et al. albumin-bound sphingosine1-phosphate has been shown to inhibit the shedding of syndecan-1 and regenerate the glycocalyx of endothelial cells. As we cannot exclude the possibility that serum free conditions affected the glycocalyx of cancer cells during cell adhesion experiments, we consider as a limitation of our study that cells were treated in serum-free solutions. Despite this limitation the enzymatic treatments were effective and caused changes in all experiments as compared to control groups also kept in serum free buffer. Moreover, in our case, the two control groups measured at 0 min and 120 min showed no difference in the zeta potential measurement (Fig. 4b).

The RWG measurements resulted in sigmoidal adhesion kinetic curves, a typical feature of active, living processes. The adhesion curves of the digested cells were sigmoidal-shaped too, which suggests that the cells are viable and adhere to the artificial surface with similar mechanisms, but reducing their glycocalyx fine-tune their adhesion properties^5,20^. The kinetic data were well fitted by the logistic differential equation. This way the maximum biosensor signal ($\Delta \lambda_{max}$) and the adhesion rate constant (*r*) could be determined in the function of the enzyme concentration. We found that enzyme treatment detuned the above parameters in a concentration dependent and regulatory manner. At low concentrations (1.25 × 10^–4^ and 6.25 × 10^–5^ U/ml) both $\Delta \lambda_{max}$ and *r* increased compared to the untreated control, meaning stronger and faster cell adhesion. In contrast, stronger digestion (above 6.25 × 10^–4^ U/ml) resulted in decreased values, meaning weaker and slower adhesion. Investigations with more concentrations supported these findings in case of HeLa cells (see SI Fig. S6). The effect of digestion was also investigated on another type of cell lines (see SI Fig. S7, S8). Interestingly, the effect on adhesion was completely missing for the control healthy cell line, and was less significant for the other type of cancer cell line. The differences we attribute to the different composition and/or structure of their glycocalyx.

To further analyze the changes in the adhesion kinetics of HeLa cells at the molecular level, we developed a kinetic model that considers the reversible formation of integrin-RGD complexes and the recruitment of integrins into the adhesion zone. Fitting the model to the measured kinetic data and analyzing the obtained parameters, we found that the enzyme treatment reduces integrin–ligand binding above 6.25 × 10^–4^ U/ml concentrations (decreased *k*_1_ with increasing ChrABC concentration). At the lowest concentrations (6.25 × 10^–5^ and 1.25 × 10^–4^ U/ml) *k*_1_ increased compared to the control (without digestion). In contrast, the recruitment rate of the integrins, *k*_3,_ decreased with increasing enzyme concentration. Enzyme treatment, however, did not influence the dissociation of integrin–ligand complexes (almost constant *k*_2_). Changes in other model parameters, such as the maximum level of bound integrin-RGD complexes, and the parameter controlling the level of integrin recruitment also suggested that at low ChrABC concentrations complex formation is facilitated, but at larger concentrations the opposite effect is dominant. Moreover, our data revealed that the HeLa cells try to compensate for the weaker and slower binding by allowing more integrins to be recruited into the adhesion zone at high enzyme concentrations, and the effect is opposite at low ChrABC concentrations.

During the two-hour experimental time we do not expect transcriptional/translational changes affecting the level of integrins. We hypothesize that the redistribution of integrins from the intracellular pool to the cell surface might be one of the mechanisms explaining the effects seen in the study. Indeed, dynamics of integrins can be amazingly fast, integrin clustering and turnover was observed within 5–10 min in focal adhesions by FRET^76^.

It is important to note, our results found at concentrations above 6.25 × 10^–4^ U/ml are well in line with previous findings on adhesion strength, but the kinetics of adhesion was not investigated previously. For example, experiments performed by Gandhi et al.^77^ on melanoma cells by digesting chondroitin sulfate and dermatan sulfate chains with chondroitinase AC and chondroitinase B fit to our result. The applied treatment inhibited the angiogenesis, proliferation, and invasion of melanoma cells. Inhibition of melanoma proliferation was achieved to a maximum of 45% with 10 U/ml chondroitinase AC treatment, whereas the same dose of chondroitinase B inhibited melanoma proliferation by only 22%. It was concluded that chondroitinase A and C also reduce integrin-preferential cell adhesion. Cell apoptosis was measured by an increase in caspase-3 activity. The previously used doses of 10 U/ml chondroitinase A and C increased caspase activity by 256%, thereby increasing the level of apoptosis compared to controls^66^. Similarly, Paszek et al. observed that the clustering of integrins is facilitated by the voluminous glycocalyx, which regulates cell growth. Short glycoproteins attached to the cell membrane show a steady distribution among integrins. However, when long synthetic glycoproteins were examined as springs, they resulted in a membrane-ECM gap where integrins could cluster, which makes it easy to from a cell adhesion site. The moderately dense glycocalyx promotes cell surface receptor clustering, receptors accumulate around the sites of adhesion, and strengthen cell adhesion^23^. Iida et al. also studiedthe effect of ChrABC and found reduced cell adhesion. They used 0.1 and 0.01 U/ml of the enzyme on human melanoma cells. They hypothesized that chondroitin-sulfate proteoglycans act on cell stability, affecting ligand binding and/or clustering. Integrins bind directly to chondroitin-sulfate and an integrin subunit contains at least one chondroitin-sulfate glycosaminoglycan binding site. This interaction directly controls the integrin adhesion property^32,33^, the adhesion strength-reducing effect of chondroitinase has been experimentally demonstrated. In another works, Delholm et al. treated human dermal fibroblast cells and human melanoma cells with 0.001 and 10 U/ml chondroitinase. At concentrations below 0.001 U/ml, the enzyme had no observable effect on the cells, but at concentrations above it inhibited cell proliferation, which also means inhibition of adhesion in the short term^65,66^.

Note, the speed of adhesion was not investigated in previous literature, but it is crucial to in-depth understand the molecular scale events during adhesion. We believe this is due to the limited time resolution of the employed methods. Moreover, while our high concentration data fits to previous findings, the observed adhesion strengthening and fastening at low concentrations needs some more explanations and implies a double-sided role of glycocalyx during cell adhesion. This effect was not reported previously, possibly due to the limited resolution of the techniques employed. This could also explain the application of relatively large enzyme concentrations in previous works.

In order to understand the complex regulatory effect of glycocalyx on cell–cell interactions in general, one should recall that one of its most prominent physiological roles is limiting the interaction of adhesion molecules (integrins) of blood cells with the endothelial cells of vessel walls, where the glycocalyx density and thickness is extremely high^78,79^. This protective function is manifested via repellent electric and mechanical interactions due to the high negative charge density and an entropic spring effect of the glycocalyx molecules^77^. Injury of the endothelial glycocalyx layer due, e.g., to toxins or inflammation may lead to serious pathological conditions, such as intravascular blood clot formation, tissue edema via enhanced permeability of the vessel wall, or dysregulated vasodilation^80,81^. These findings suggest that a dense glycocalyx layer composed of long polysaccharide chains can actually hamper adhesion of cells, which can be overcome by mild enzymatic digestion of the layer. Given that cancer cells are known to overexpress glycocalyx, we assume that the faster and increased adhesion found at low chondroitinase concentrations can be attributed to the reduction of this spacer effect of dense glycocalyx.

On the contrary, a moderately dense glycocalyx layer is suggested to promote clustering of integrins on cell surfaces^23^. Paszek et al. traces this phenomenon back primarily to a delicate, dynamic balance of nanomechanical interactions of two types of glycocalyx components (“stiff beams” and “flexible chains”) with each other, the deformable cell membrane and the external ligands^77^. Based on our earlier findings on the Hofmeister-phenomenon of kosmotropic salting-out^82–84^, we raise a complementary argumentation. Namely, dominant side chains of the glycocalyx (e.g., the sulfate and carboxyl groups of heparan or chondroitin sulfate, and hyaluronic acid, respectively) are considered highly kosmotropic agents^85^, ordering adjacent water molecules stronger than integrin side chains do (containing, amongst others, non-kosmotropic beta-sheet structures). This difference in H-bonding propensity with water molecules should make the glycocalyx-forming molecules and the integrins separated into two phases, such as a two-dimensional analogue of kosmotropic salting-out^85,86^. Our finding that the integrin-RGD dissociation is not affected by the digestion level of glycocalyx is fully supporting the above theory, since glycocalyx components are excluded from the adhesion sites, less influencing in this way its further ligand binding properties. In any way, the clustering effect of glycocalyx on integrins should be weakened by further digesting the polysaccharide layer, as it is shown by our experimental data, as well.

Using the obtained kinetic rate constants, we determined the enzyme concentration dependence of the dissociation constant of integrin-RGD binding, directly in live cells, without employing any labelling. This “kinetic” dissociation constant (${K_{d}^{k}}$) was compared to a “static” dissociation constant (${K_{d}^{s}}$) obtained by an alternative way. In the latter case, we measured the maximum level of adhesion signals on RGD density tuned surfaces and assumed a monovalent binding model between the integrins and their RGD ligands.

Even if two completely different strategies were employed to obtain the dissociation constant, they differed by only an order of magnitude, the ${K_{d}^{s}}$ being larger. We revealed that at high enzyme concentrations the two values are even more close to each other. There are several possible explanations of this interesting result. One can assume that the kinetic parameters regulating the molecular scale events are not constant over time. ${K_{d}^{k}}$ is clearly a time average, but ${K_{d}^{s}}$ refers to the end state of values. Another possible explanation is that the RGD surface density also plays an important role in glycocalyx regulated adhesion processes. ${K_{d}^{s}}$ in this respect is an averaged value calculated from a broad range of RGD densities, while ${K_{d}^{k}}$ came only from the 50% RGD density, a single point. We believe, further targeted experiments are needed to investigate this result in more detail.

Our results imply that if the cell is able to regulate its glycocalyx content, it will be able to control and fine tune its adhesion properties. Cancer cells, e.g., can choose between adhesion or migration, according to the external conditions by regulating their glycocalyx content. Overall, the above results suggest a regulatory mechanism of glycocalyx in cancer cell adhesion, both the strength and speed of adhesion is regulated by a sophisticated manner. The developed methodology can be easily adapted to other types of cells or other types of enzymatic digestions, opening new pathways to study the effect of glycocalyx on the kinetics of cell adhesion. Using kinetic modelling and the high-resolution label-free data one can depict the time dependent changes of biomolecular parameters of living cells, which is challenging by employing traditional methods. Moreover, the discovered cell type specific effects potentially open the way for novel type of treatments, selectively targeting cells with a specific glycocalyx composition or structure.

## Supplementary Information

Supplementary Information.
