# Supplementary material for: Glycocalyx regulates the strength and kinetics of cancer cell adhesion revealed by biophysical models based on high resolution label-free optical data
Source: Sci Rep. 2020 Dec 30;10:22422. doi: 10.1038/s41598-020-80033-6 (PMC7773743; doi:10.1038/s41598-020-80033-6)
Supplement: Supplementary file 1 — Supplementary Information. [file 41598_2020_80033_MOESM1_ESM.docx]

**Supplementary Information**

for

**Glycocalyx regulates the strength and kinetics of cancer cell adhesion revealed by biophysical models based on high resolution label-free optical data**

Nicolett Kanyo^a^, Kinga Dora Kovacs^a^, Andras Saftics^a^ , Inna Szekacs^a^, Beatrix Peter^a^, Ana R. Santa-Maria^b,c,d^, Fruzsina R. Walter^b,d^, András Dér^b^, Mária A. Deli^b^, Robert Horvath^a,*^

^a^ Nanobiosensorics Momentum Group, Institute of Technical Physics and Materials Science, Centre for Energy Research, Konkoly-Thege M. út 29-33, H-1120 Budapest, Hungary

^b^ Institute of Biophysics, Biological Research Centre, Temesvári krt. 62., H-6726, Szeged, Hungary

^c^ Doctoral School of Biology, University of Szeged, Közép fasor 52., H-6726, Szeged, Hungary

^d^ Department of Biotechnology, University of Szeged, Közép fasor 52., H-6726, Szeged, Hungary

* Corresponding author:

Robert Horvath

email: [horvathr@mfa.kfki.hu](mailto:horvathr@mfa.kfki.hu), [horvath.robert@energia.mta.hu](mailto:horvath.robert@energia.mta.hu)

**Materials and methods**

**Cell cultures**

Preosteoblast cell line MC3T3-E1 (99072810, ECACC General Collection) was maintained in tissue culture polystyrene Petri dishes (Sarstedt, Germany) in a humidified incubator (37 °C, 5% CO_2_) in α-modified minimal essential medium, supplemented with 10% fetal bovine serum (Biowest SAS, France), 2 mM l-glutamine, 100 U/ml penicillin, 100μg/ml streptomycin solution, and 0.25 μg/ml amphotericin B. Cells were passaged by using 0.05% (w/v) trypsin and 0.02% (w/v) EDTA solution (Merck, Germany)

MCF-7 cells, a breast cancer cell line (ATCC), were cultured in tissue culture polystyrene Petri dishes (Sarstedt, Germany) in a humidified incubator (37 °C, 5% CO_2_) in 50% of DMEM (Gibco, 21885108; low glucose content: 1 g/l) and in 50% of DMEM/F12 (Gibco, 31331-028; high glucose content: 4.5 g/l) and 10% FBS (Sera Plus, Pan Biotech, P30-3702). Cells were passaged by using 0.05% (w/v) trypsin and 0.02% (w/v) EDTA solution (Merck, Germany).

**MTT cell viability assay**

To test the effect of chondroitinase ABC (ChrABC) treatment on cell viability we performed an MTT dye reduction assay. Living and metabolically active cells convert the yellow MTT dye (3-(4,5-dimethylthiazol-2-yl)-2,5-diphenyltetrazolium bromide) to purple formazan crystals. For this method we cultured the HeLa cells on 96-well plates (Corning Costar, USA) at a cell number of 1.5 × 10^4^ / well. Confluent cultures were treated with with ChrABC concentrations between 1.00 × 10^-5^ to 1.25 × 10^-1^ U/ml for 60 min and with 1.25 U/ml for 60 and 120 min with relevant controls treated only with HBSS-HEPES buffer at room temperature. Triton X-100 detergent was used as a 100% cytotoxic agent. After the treatments the MTT solution (0.5 mg/ml) was added to the cells in phenol red free DMEM/F12 and cells were incubated for 2 h at 37 °C. Formazan crystals produced by the metabolically active viable cells were dissolved with concentrated dimethyl sulfoxide, and absorbance was measured at 592 nm by a multiwell microplate reader (Fluostar Optima, BMG Labtechnologies, Germany). Cytotoxicity was calculated as percentage of the control where the maximum dye conversion was observed.

**Wheat germ agglutinin glycocalyx staining and confocal microscopy**

For the wheat germ agglutinin staining cells were prepared similarly to the chondroitin sulfate staining (Fig. 2). HeLa, MCF-7 and MC3T3-E1 cells were cultured on poly-L-lysine coated glass cover slips for 2-3 days and after the cultures covered the available surface cells were treated with 1.25 U/ml ChrABC in HEPES HBSS buffer for 0 and 60 min at room temperature. After treatment cells were fixed with 1% paraformaldehyde (PFA)-PBS for 15 min at room temperature. Cells were not permeabilized to ensure only surface labeling. Labeling of *N*-acetylneuraminic (sialic) acid and *N*-acetyl-d-glucosamine residues within the glycocalyx was done using wheat germ agglutinin lectin (WGA) conjugated with Alexa Fluor 488 (Invitrogen, W11261). Cells were incubated with the lectin solution (5 µg/ml in PBS) for 10 min at room temperature (Betteridge et al., 2017). Pictures were taken with an Olympus FV1000 confocal microscope at random positions, at least 5 images/cover slip. Treatments were performed in triplicates (total of 15 pictures / group). Fluorescent images were analyzed for staining intensity using the FIJI (ImageJ) software.

**Polymer solutions for coating the biosensor surfaces**

The synthetic copolymers, poly(L-lysine)-*graft*-poly(ethylene glycol) (PLL-*g*-PEG, [PLL(20)-g(3.5)-PEG(2)]) (hereafter PP) and its RGD-functionalized counterpart, PLL-*g*-PEG/PEGGGGGYGRGDSP (PLL-*g*-PEG-RGD [PLL(20)-g(3.5)-PEG(2)/PEG(3.4)-RGD]) (hereafter PPR) and PLL-*g*-PEG-(DBCO-Mal)-CKK-(Acp)-(Acp)-(Acp)-GRGDS (PLL(20)-g(3.5)-PEG(2)/PEG(3.5)-RGD) (hereafter PP-DBCO-R)) were obtained as powders from SuSoS AG, Dübendorf, Switzerland.

The materials were stored at -20 °C until use. Each powder was then dissolved in 10 mM HEPES at pH 7.4 to make stock solutions with a concentration of 1.0 mg/ml and sterile filtered. Coating solution with different concentration of RGD-motifs and PLL-*g*-PEG were prepared by mixing the two 1 mg/ml stock solutions (hereafter PP:PPR). HeLa and MC3T3-E1 cells were measured on 50% PP: PPR surface, MCF-7 cells on 50% PP-DBCO-R surface for cell adhesion measurements.

**Results**

**MTT cell viability assay**

The effect of ChrABC on the metabolic activity of HeLa cells was studied at 1.00 × 10^-5^ to 1.25 × 10^-1^ U/ml concentrations for 60 min and with 1.25 U/ml for 60 and 120 min with time-matching untreated controls **(Figure S1)**. ChrABC treatment did not cause any change in the viability of HeLa cells at neither concentration examined in the tested time points. Cells survived well at room temperature in the HEPES HBSS buffer as well reflecting a proper environment for our studies. Triton X-100 detergent, used as a reference molecule to induce toxicity, on the other hand showed an almost 100% viability decrease showing the validity of the test.


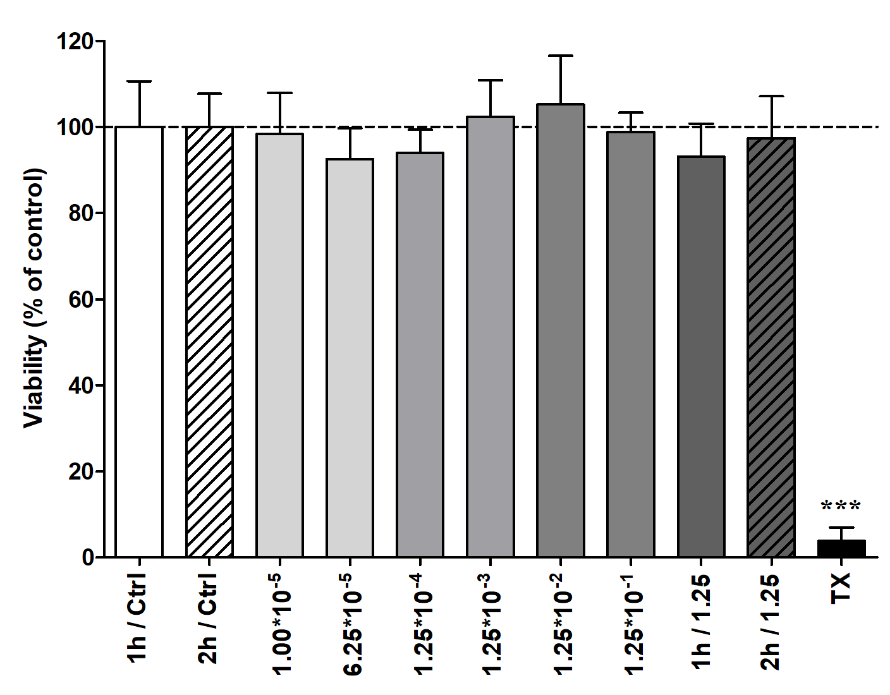


**Figure S1.** The effects of chondroitinase ABC on cell viability measured by MTT assay on HeLa cells. Treatments were performed for 1 h and 2 h with concentrations of 1.00 × 10^-5^ to 1.25 × 10^-1^ U/ml. Ctrl: control. TX: Triton X-100 detergent. Values of each group are presented as mean ± SD, n = 4–12. Data were analyzed by one-way ANOVA followed by Bonferroni post-test. ***, p < 0.001, compared to the 1 h control.

**Time dependency of ChrABC enzyme treatment on surface chondroitin sulfate immunostaining of HeLa cells**

We performed a test to determine the effects of ChrABC treatment on the HeLa cells chondroitin sulfate immunostaining at 1.25 U/ml at different timepoints **(Figure S2)**. The fluorescent intensity of chondroitin sulfate immunostaining of HeLa cells treated for 30, 60 and 120 min was evaluated. Staining intensity of chondroitin sulfate decreased already after 30 min of ChrABC treatment, which effect persisted until the 120 min time point. For our experiments we chose therefore the 60 min time point to see a complete ChrABC activity effect in accordance with enzyme kinetics data (Yamagata et al., 1968)


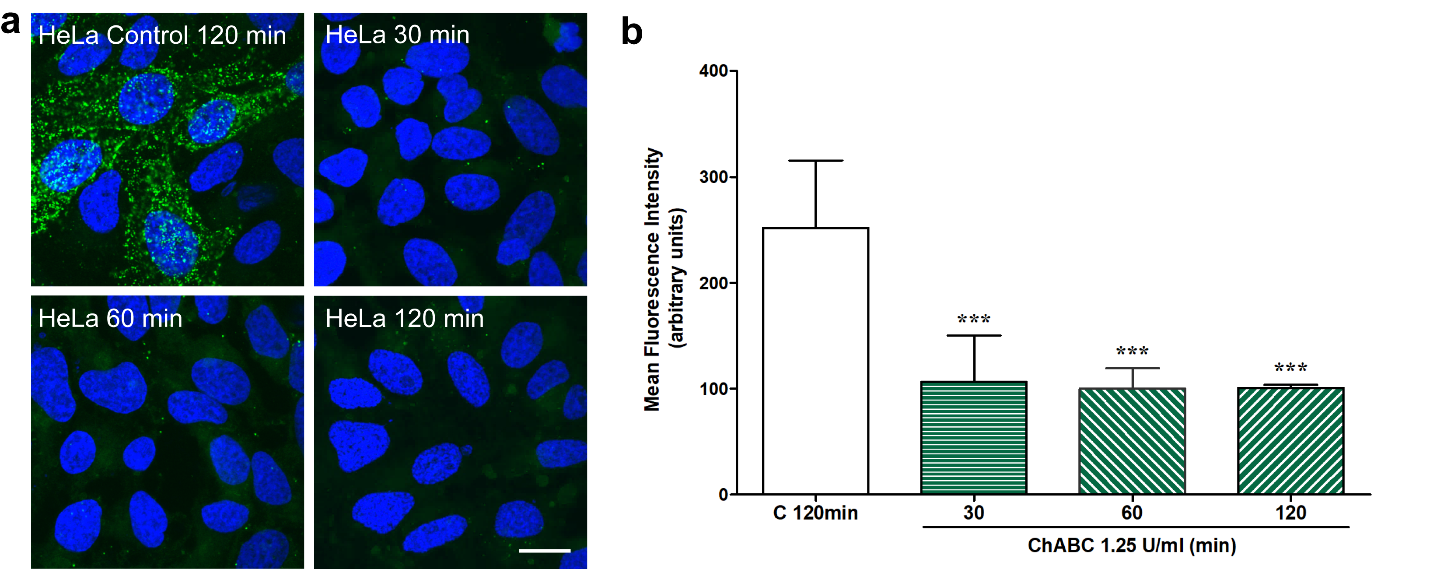


**Figure S2.** **(A)** Representative images of the chondroitin sulfate immunostaining on HeLa cells after 30, 60 and 120 min 1.25 U/ml ChrABC treatment. Scale bar: 50 µm. **(B)** Intensity evaluation of the chondroitin sulfate immunostainings. n=15 images/treatment groups, ***, p<0.001 compared to the untreated control. One-way ANOVA with Bonferroni post-test.

**Wheat germ agglutinin glycocalyx staining of HeLa cells**

Besides the chondroitin sulfate immunostaining, we performed wheat germ agglutinin (WGA) lectin staining to visualize different components of the glycocalyx. We stained the HeLa cells **(Figure S3A)** with WGA-Alexa488, which labels the N-acetylneuraminic (sialic) acid and N-acetyl-D-glucosamine residues within the glycocalyx. We observed that the intensity of the staining did not change at either of the treatment time-points **(Figure S3B)**. This shows, that the ChrABC treatment reduces the chondroitin sulfate coverage from the cell surface of HeLa cells, but leaves other glycocalyx components less affected. We also performed a 3D analysis of the staining to demonstrate that the WGA staining can be observed at the cell surface of HeLa cells. This was ensured during the staining protocol by fixing but not permeabilizing the cells before staining with WGA lectin **(Figure S3C).**


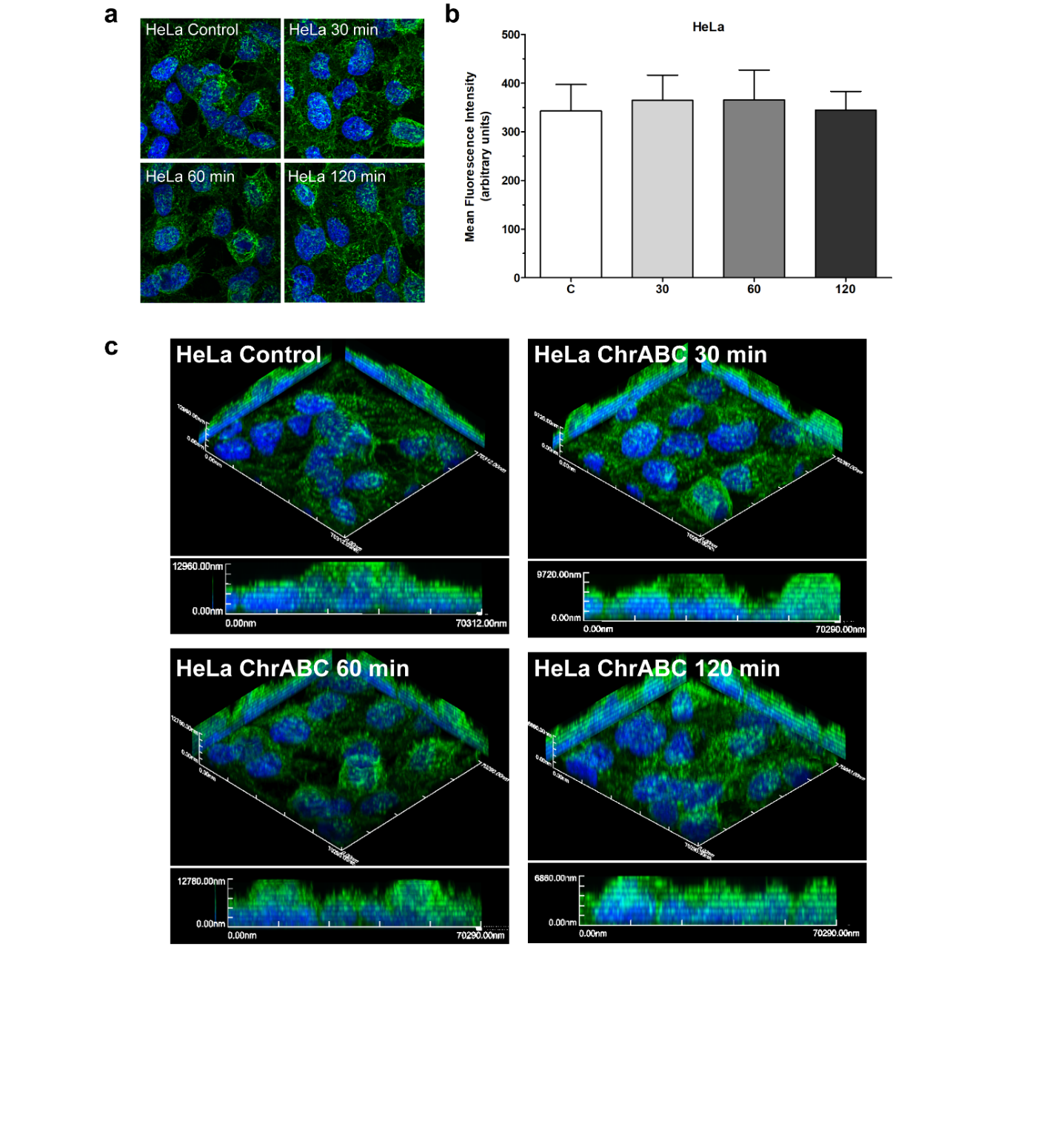


**Figure S3.** Wheat germ agglutinin staining of HeLa cells. **(A)** Representative images of the WGA staining on HeLa cells after 30, 60 and 120 min ChrABC treatment (1.25 U/ml). Scale bar: 20 µm. **(B)** Intensity analysist of the fluorescent WGA staining. n=15 images/treatment groups, one-way ANOVA with Bonferroni post-test. C: control. No significant differences were found. **(C)** 3D images of the representative pictures. Analysis was performed using the FV-1000 Olympus software.

**Chondroitin sulfate immunostaining and WGA lectin labeling of MCF-7 and MC3T3-E1 cells treated with ChrABC**

We also performed chondroitin immunostaining on the MCF-7 breast cancer cell line and the MC3T3-E1 non-tumor preosteoblast cell line to characterize their reaction to the ChrABC enzyme digestion **(Figure S4A and B)**. We chose the 60 min time point and the 1.25 U/ml ChrABC treatment concentration according to previous experiments. We observed a decrease in the chondroitin sulfate immunostaining intensity in the MCF7 cell line after the enzyme digestion, but saw no change in the case of the MC3T3 cells. The cell surface glycocalyx was labeled with WGA staining for both cell types **(Figure S4C and D)**. As in the case of HeLa cells, we did not see any change in the staining intensity after the ChrABC treatment.


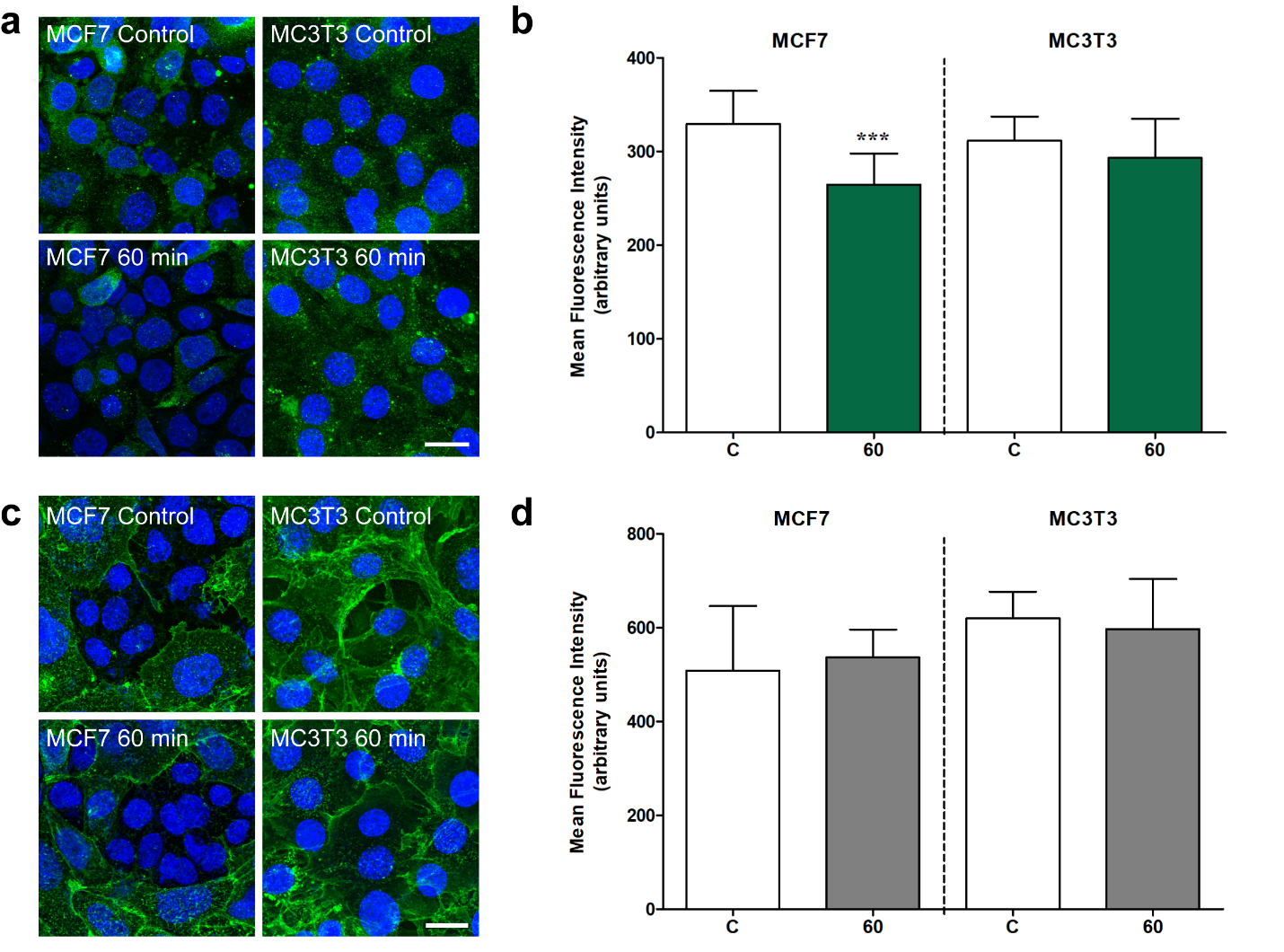


**Figure S4.** Chondroitin sulfate immunostaining and WGA lectin labeling of MCF-7 and MC3T3-E1 cell lines. **(A)** Representative images after 60 min ChrABC treatment (1.25 U/ml). Scale bar: 20 µm. **(B)** Intensity analysis of the fluorescent chondroitin sulfate immunostaining. n=15 images/treatment groups, one-way ANOVA with Bonferroni post-test. C: control. ***, p<0.001 compared to the untreated control. **(C)** Representative images of the WGA staining after 60 min ChrABC treatment (1.25 U/ml). Scale bar: 20 µm. **(D)** Intensity measurement of the fluorescent WGA staining. n=15 images/treatment groups, one-way ANOVA with Bonferroni post-test. C: control. No significant differences were found.

**Zeta potential measurement of ChrABC treated MCF-7 and MC3T3-E1 cells**

For the zeta potential measurements, cells in suspension were treated with 1.25 U/mL of ChrABC in HEPES HBSS for 60 min. Control (untreated) cells were measured twice: one control group was measured directly after cell counting, at the beginning of the experiment (C, 0 min), while the other control group was incubated in HEPES HBSS for 60 min without any enzymatic treatment (C, 60 min). Interestingly, we obtained the result that the zeta potential of MCF-7 cells was significantly reduced by ChrABC treatment, and the zeta potential of MC3T3-E1 cells was significantly increased after 60 min of treatment. We cannot fully explain these changes. We hypothesize that in specific cell types (cancer vs. non-cancer) the adhesion properties, zeta potential values and glycocalyx changes may be regulated by different mechanisms and/or by the unique glycocalyx composition and its sensitivity to enzyme digestion. The basal zeta potential of the MCF-7 control cells was -8.6 mV (**Figure S5**). ChrABC treatment significantly decreased the zeta potential after 60 min treatment to -9.7 mV suggesting that other glycocalyx components might contribute to a greater degree to the negative surface charge than chondroitin sulfate. The MC3T3-E1 cells showed a negative baseline zeta potential, -11.7 mV, similar to that of HeLa cells (‑11.9 mV). The ChrABC treatment decreased the absolute value of the zeta potential to -10.4 mV, which effect was less strong, but close to the HeLa cells’ reaction to the enzyme digestion. This experiment demonstrates that there are big differences in baseline surface charge values between different cell lines depending on their function, origin and most probably glycocalyx composition.


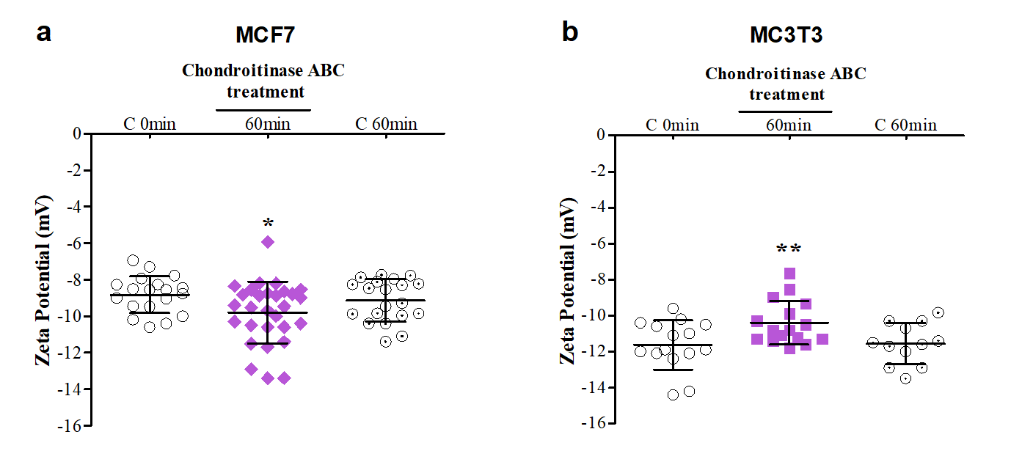


**Figure S5.** Zeta potential measurement after ChrABC treatment (1.25 U/ml) on MCF-7 **(A)** and MC3T3-E1 **(B)** cells. Measurements were performed after 60 min incubation with the enzyme. C: control. Control measurement was performed both at the beginning and at the end of the experiment with separate biological parallels. Three biological parallels were measured (n=13-29). *, p<0.05; **, p<0.01 compared to the 0 min control. One-way ANOVA with Bonferroni post-test.

**Normalized adhesion response of the cancer cell lines HeLa and MCF-7, and the preosteoblast MC3T3-E1 cell line after chondroitinase ABC digestion**

In case of HeLa cells, cell adhesion decreases at high enzyme concentrations (greater than 0.5×10^- 3^ U/ml) and an increase in maximum cell adhesion signal was observed at lower concentrations **(Figure S6)**.


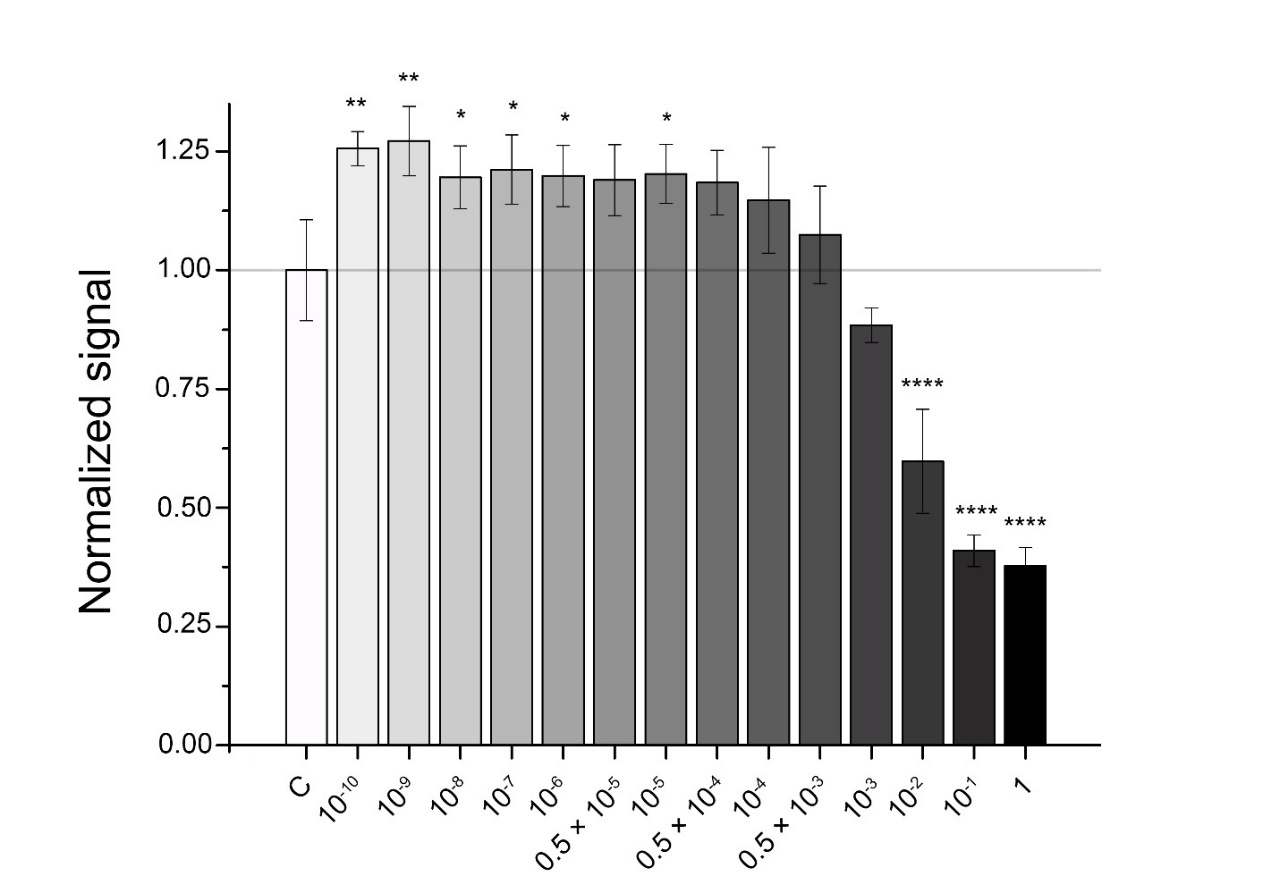


**Figure S6.** Normalized adhesion response for HeLa cells with ChrABC (U/ml) digestion. *, p<0.1, **, p<0.01, ****, p <0.0001 (C: control, 0 U/mL)

The MCF-7 cell line is found to be more resistant to ChrABC treatment, the adhesion signal only slightly changes due to the treatment. Significant change with (p<0.01) could only be achieved with 1 U / ml ChrABC treatment **(Figure S7)**. At the highest employed ChrABC concentration removal of chondroitin sulfate from this cell line was confirmed by immunoistaining (**Figure S4**). Interestingly, this enzyme treatment did not decrease the absolute value of the zeta potential on this breast cancer cell line, in contrast to the other two cell types investigated. This may indicate a different glycocalyx composition responsible for the observed differences as compared to HeLa cells.


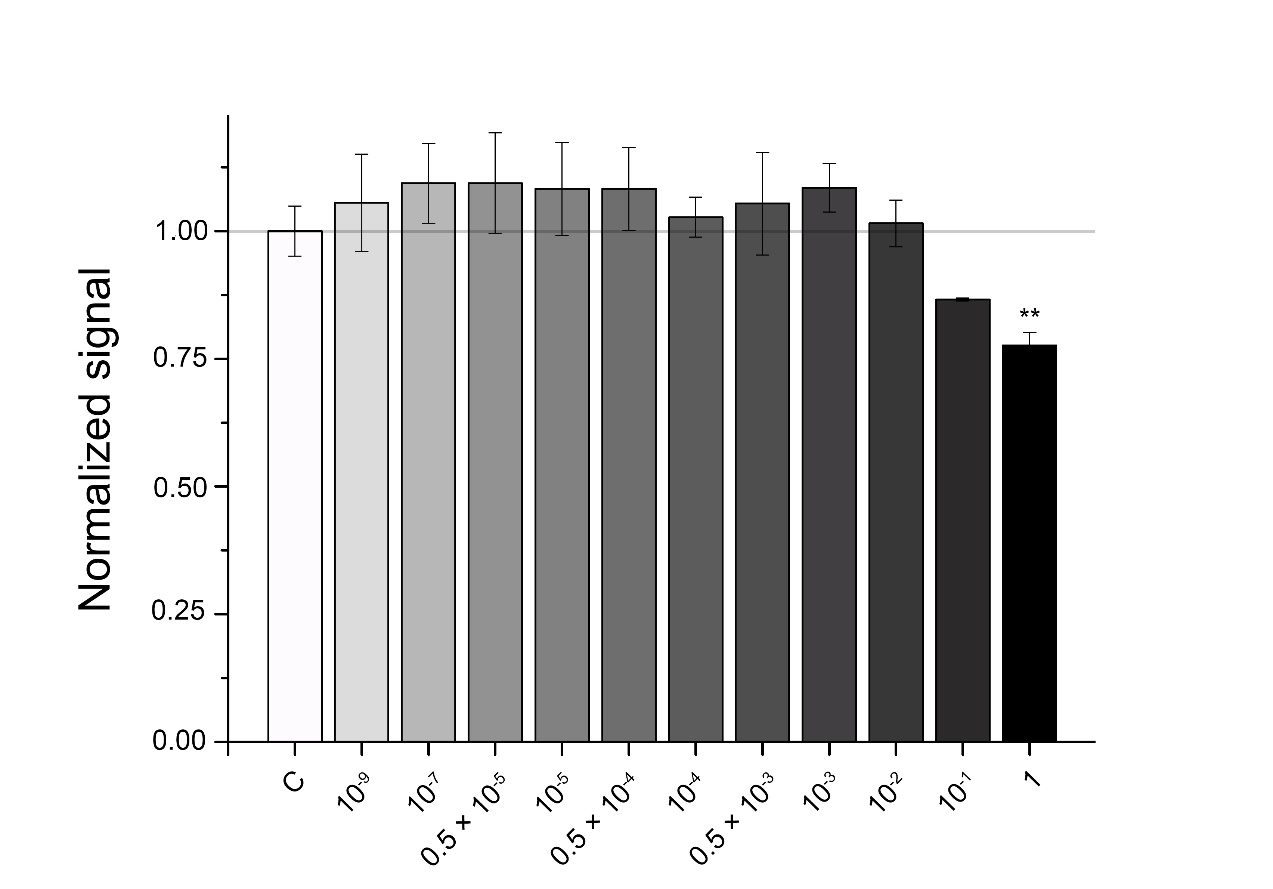


**Figure S7.** Normalized adhesion response for MCF-7 cells with ChrABC (U/ml) digestion **, p<0.01. (C: control, 0 U/mL)

It can be clearly seen that ChrABC had no significant effect on the preosteoblast cell adhesion at none of the enzyme concentrations **(Figure S8**). Similarly, no change was observed for glycocalyx staining in this cell line after ChrABC treatment (**Figure S4**).


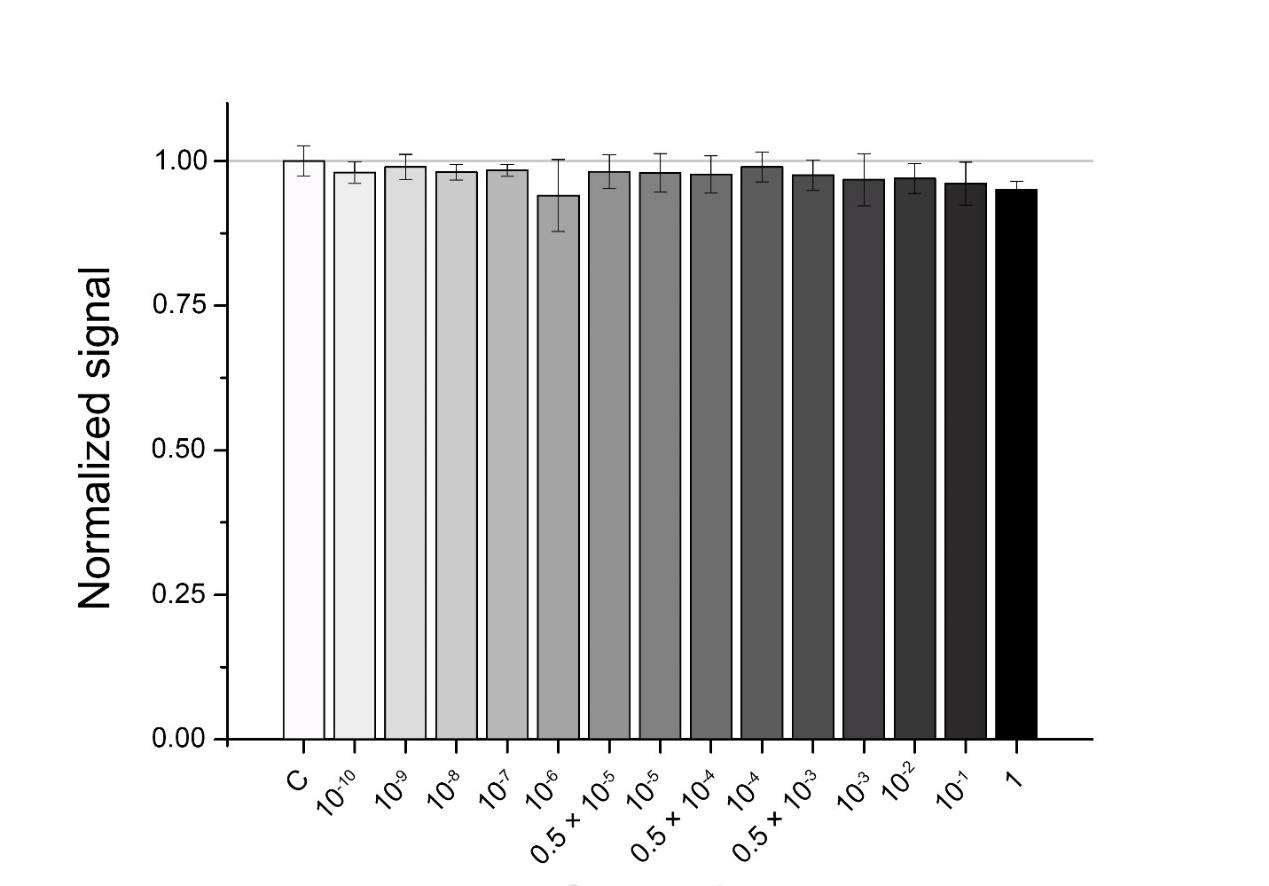


**Figure S8.** Normalized adhesion response for preosteoblast (MC3T3-E1) cells with ChrABC (U/ml) digestion. (C: control, 0 U/mL)

One possible reason for this may be that the MC3T3-E1 is a non-tumor cell line and might have a different glycocalyx composition as cancer cells, which have particularly rich surface sugar layers (Xu et al., 2016; Hollingsworth and Swanson, 2004). Others have described that CS overexpression in MC3T3-E1 cells increased adhesion of the cells to N-cadherin and cadherin-11, and promoted osteoblast differentiation (Koike et al., 2012). Thus, it is likely that the enzyme has an effect on this cell type, but not on the adhesion to RGD motifs displaying surfaces (or on WGA or chondroitin sulfate cell surface glycocalyx staining). This potential enzymatic effect was revealed by our data on the decrease of the absolute value of the zeta potential (**Figure S5**).

**References**

Betteridge KB, Arkill KP, Neal CR, Harper SJ, Foster RR, Satchell SC, Bates DO, Salmon AHJ. Sialic acids regulate microvessel permeability, revealed by novel in vivo studies of endothelial glycocalyx structure and function. J Physiol. 2017 Aug 1;595(15):5015-5035. doi: 10.1113/JP274167. PMID: 28524373; PMCID: PMC5538239.

Hollingsworth MA, Swanson BJ. Mucins in cancer: protection and control of the cell surface. Nat Rev Cancer. 2004 Jan;4(1):45-60. doi: 10.1038/nrc1251. PMID: 14681689.

Koike T, Izumikawa T, Tamura J, Kitagawa H. Chondroitin sulfate-E fine-tunes osteoblast differentiation via ERK1/2, Smad3 and Smad1/5/8 signaling by binding to N-cadherin and cadherin-11. Biochem Biophys Res Commun. 2012 Apr 13;420(3):523-9. doi: 10.1016/j.bbrc.2012.03.024. Epub 2012 Mar 13. PMID: 22440395.

Xu GK, Qian J, Hu J. The glycocalyx promotes cooperative binding and clustering of adhesion receptors. Soft Matter. 2016 May 18;12(20):4572-83. doi: 10.1039/c5sm03139g. PMID: 27102288.

Yamagata T, Saito H, Habuchi O, Suzuki S. Purification and properties of bacterial chondroitinases and chondrosulfatases. J Biol Chem. 1968 Apr 10;243(7):1523-35. PMID: 5647268.
